# Covalent *Plasmodium falciparum*-selective proteasome inhibitors exhibit a low propensity for generating resistance *in vitro* and synergize with multiple antimalarial agents

**DOI:** 10.1371/journal.ppat.1007722

**Published:** 2019-06-06

**Authors:** Barbara H. Stokes, Euna Yoo, James M. Murithi, Madeline R. Luth, Pavel Afanasyev, Paula C. A. da Fonseca, Elizabeth A. Winzeler, Caroline L. Ng, Matthew Bogyo, David A. Fidock

**Affiliations:** 1 Department of Microbiology and Immunology, Columbia University Irving Medical Center, New York, NY, United States of America; 2 Department of Pathology, Stanford University School of Medicine, Stanford, CA, United States of America; 3 Division of Host-Microbe Systems & Therapeutics, Department of Pediatrics, University of California San Diego, School of Medicine, San Diego, CA, United States of America; 4 MRC Laboratory of Molecular Biology, Cambridge Biomedical Campus, Cambridge, United Kingdom; 5 Division of Infectious Diseases, Department of Medicine, Columbia University Irving Medical Center, New York, NY, United States of America; Francis Crick Institute, UNITED KINGDOM

## Abstract

Therapeutics with novel modes of action and a low risk of generating resistance are urgently needed to combat drug-resistant *Plasmodium falciparum* malaria. Here, we report that the peptide vinyl sulfones WLL-vs (WLL) and WLW-vs (WLW), highly selective covalent inhibitors of the *P. falciparum* proteasome, potently eliminate genetically diverse parasites, including K13-mutant, artemisinin-resistant lines, and are particularly active against ring-stage parasites. Selection studies reveal that parasites do not readily acquire resistance to WLL or WLW and that mutations in the β2, β5 or β6 subunits of the 20S proteasome core particle or in components of the 19S proteasome regulatory particle yield only <five-fold decreases in parasite susceptibility. This result compares favorably against previously published non-covalent inhibitors of the *Plasmodium* proteasome that can select for resistant parasites with >hundred-fold decreases in susceptibility. We observed no cross-resistance between WLL and WLW. Moreover, most mutations that conferred a modest loss of parasite susceptibility to one inhibitor significantly increased sensitivity to the other. These inhibitors potently synergized multiple chemically diverse classes of antimalarial agents, implicating a shared disruption of proteostasis in their modes of action. These results underscore the potential of targeting the *Plasmodium* proteasome with covalent small molecule inhibitors as a means of combating multidrug-resistant malaria.

## Author summary

The spread of artemisinin-resistant *Plasmodium falciparum* malaria across Southeast Asia creates an imperative to develop new treatment options with compounds that are not susceptible to existing mechanisms of antimalarial drug resistance. Recent work has identified the *P*. *falciparum* proteasome as a promising drug target. Here, we report potent antimalarial activity of highly selective vinyl sulfone-conjugated peptide proteasome inhibitors, including against artemisinin-resistant *P*. *falciparum* early ring-stage parasites that are traditionally difficult to treat. Unlike many advanced antimalarial candidates, these covalent proteasome inhibitors do not readily select for resistance. Selection studies with cultured parasites reveal infrequent and minor decreases in susceptibility resulting from point mutations in components of the 26S proteasome, which we model using cryo-electron microscopy-based structural data. No parasites were observed to be cross-resistant to both compounds; in fact, partial resistance to one compound often created hypersensitivity to the other. We also document potent synergy between these covalent proteasome inhibitors and multiple classes of antimalarial agents, including dihydroartemisinin, the clinical candidate OZ439, and the parasite transmission-blocking agent methylene blue. Proteasome inhibitors have significant promise as components of novel combination therapies to treat multidrug-resistant malaria.

## Introduction

*Plasmodium falciparum* malaria threatens 40% of the world’s population, resulting in an estimated 220 million cases annually. Of the ~435,000 annual malaria deaths worldwide, the majority occur in African children below the age of five [1]. The treatment of *P*. *falciparum* malaria is vitally dependent on artemisinin (ART) derivatives, exceptionally fast-acting antimalarial endoperoxides that were adopted globally nearly two decades ago as the core components of ART-based combination therapies (ACTs) [2,3]. The rapid sweep across Asia of parasites that display slow rates of clearance following treatment with the ART derivative artesunate or with ACTs (referred to herein as ART-resistant or ART-R parasites) has created a significant need for new treatments that can combat resistance [4–6].

Genomic, clinical epidemiologic, and genetic studies provide compelling evidence that ART resistance is mediated primarily by mutations in the *P*. *falciparum* K13 protein [4,7–9]. K13 is a member of the BTB-Kelch family that can mediate interactions between certain E3 ubiquitin ligase complexes and substrates targeted for degradation by the ubiquitin-proteasome system (UPS) [10,11]. In this protein family, the upstream BTB domain typically binds the E3 ligase complex, which transfers ubiquitin moieties to the substrate protein, while the C-terminal six-bladed β-propeller Kelch domain binds the substrate itself, conferring specificity. While the function of *P*. *falciparum* K13 is uncharacterized, its Kelch domain harbors single point mutations that are associated with ART resistance, including the C580Y mutation that is predominant in Southeast (SE) Asia and the R539T mutation that confers high-level resistance *in vitro* [7–9,12,13].

Several experimental studies support a connection between ART, K13 and the UPS. Transcriptional profiling of ART-R SE Asian field isolates earlier revealed an upregulation of components of the UPS in K13 mutant isolates, including several proteasome subunits [14]. Additionally, several genes associated with protein folding and trafficking to or from the ER were upregulated, including subunits of two putative chaperone complexes—the *Plasmodium* reactive oxidative stress complex (PROSC) and the TCP-1 ring complex (TRiC). These results suggest that ART induces widespread protein damage, activating cell stress and proteostasis response pathways, and that ART-R K13 mutant parasites may possess an intrinsic ability to combat drug-induced alkylation via the repair or degradation of damaged proteins and other biomolecules.

In support of this hypothesis, a K13 mutant Cambodian *P*. *falciparum* isolate (PL7) was reported to show lower amounts of ubiquitinated proteins following exposure to a brief pulse of ART as compared to a K13 wild-type (WT) Cambodian isolate (PL2) [15]. However, those data were obtained from non-isogenic field isolates [14,15], and thus the differential responses could be attributable to K13, or to other genetic differences. The ART metabolite DHA was also recently reported to disrupt *P*. *falciparum* proteasome-mediated protein degradation, in addition to generating a backlog of damaged proteins, thereby overwhelming the UPS with substrates. Treating parasites with a translation inhibitor or with an inhibitor of E1 ubiquitin-activating enzymes protected cells against the effects of DHA, which was attributed to the generation of fewer UPS substrates [16].

These observations have highlighted the proteasome as a novel and promising drug target for combatting ART-R *P*. *falciparum* infections. This multi-subunit 26S complex consists of a 20S core catalytic subunit capped by 19S regulatory complexes [17]. In eukaryotes, the proteasome contributes to diverse cellular processes ranging from cell cycle progression to apoptosis via its tightly regulated degradation of ubiquitin-tagged substrates [18]. Initial studies of the *Plasmodium* proteasome revealed that the irreversible inhibitor lactacystin blocked sporozoite development into exoerythrocytic forms and inhibited *P*. *falciparum* asexual blood stage cell cycle progression [19]. The proteasome inhibitor epoxomicin was also shown to be potent against transmissible *P*. *falciparum* stage V gametocytes and to block oocyst development within the mosquito midgut [20]. Synergy between epoxomicin and DHA was reported in *P*. *falciparum*, as was *in vivo* synergy between the related epoxyketone carfilzomib and DHA in the rodent parasite *P*. *berghei* [15]. These previous-generation inhibitors were not viable as antimalarial therapeutics, however, due to high levels of toxicity resulting from inhibition of the host proteasome.

Recent studies with a variety of scaffolds have sought to improve selectivity for the *P*. *falciparum* proteasome [21–25]. Compounds resulting from these efforts include the covalent peptide vinyl sulfone inhibitors WLL-vs and WLW-vs (referred to herein as WLL and WLW), which are highly selective for the parasite proteasome over the human enzyme [21]. WLL also effectively cleared a *Plasmodium chabaudi* rodent malaria parasite infection without significant toxicity to the host [21]. These compounds exploit the parasite’s preference for bulky aromatic substrates in various positions of the β2 and β5 subunit active sites of the proteasome [21,26]. WLL and WLW potently inhibit the β2 and β5 subunits or the β2 subunit alone, respectively. WLW was shown to have strong activity against the ART-R PL7 isolate and the ART-sensitive (ART-S) PL2 isolate, and showed synergy with DHA against PL7 [21]. PL7 was ~two-fold more sensitive than PL2, suggesting a possible impact of the *K13* genotype.

Given the threat of multidrug-resistant *P*. *falciparum* malaria and the recognized need to delineate the risk for parasite resistance to preclinical antimalarial candidates, we have interrogated mechanisms of resistance in the peptide vinyl sulfone inhibitors WLL and WLW in both ART-S and ART-R parasites. Mutations conferring low-grade resistance were characterized through activity-based profiling of the proteasome beta subunits and molecular modeling based on the known cryo-electron microscopy-based structure of the *P*. *falciparum* 20S proteasome [21]. We also screened for antimalarial agents that could overcome existing antimalarial resistance mechanisms when combined with WLL or WLW. Our results, including the identification of a unique stage-specificity profile for these two proteasome inhibitors, highlight the promising features of this class of compounds.

## Results

### The *Plasmodium*-selective proteasome inhibitors WLL and WLW are potent against *P*. *falciparum* SE Asian parasites regardless of their *K13* genotype

To examine whether the potencies of the *Plasmodium*-specific proteasome inhibitors WLL and WLW were impacted by mutations in *K13*, we tested these two compounds against sets of isogenic *P*. *falciparum* lines that express WT or mutant forms of this gene. Assays included the Cam3.II (Cambodia) parental line that expresses the K13 R539T variant and that was culture-adapted in 2010, as well as the V1/S (Vietnam) parental line that is K13 WT and that was culture-adapted in the 1970s, prior to the use of ACTs. These parental lines had previously been edited using zinc-finger nucleases to express the K13 WT or C580Y alleles in Cam3.II parasites, and K13 WT, R539T or C580Y in V1/S parasites (**S1 Table**; [9]). These lines are referred to herein as Cam3.II K13^R539T^ (the unedited parental line), Cam3.II K13^WT^, Cam3.II K13^C580Y^, V1/S K13^WT^, V1/S K13^R539T^, and V1/S K13^C580Y^.

**Fig 1:**
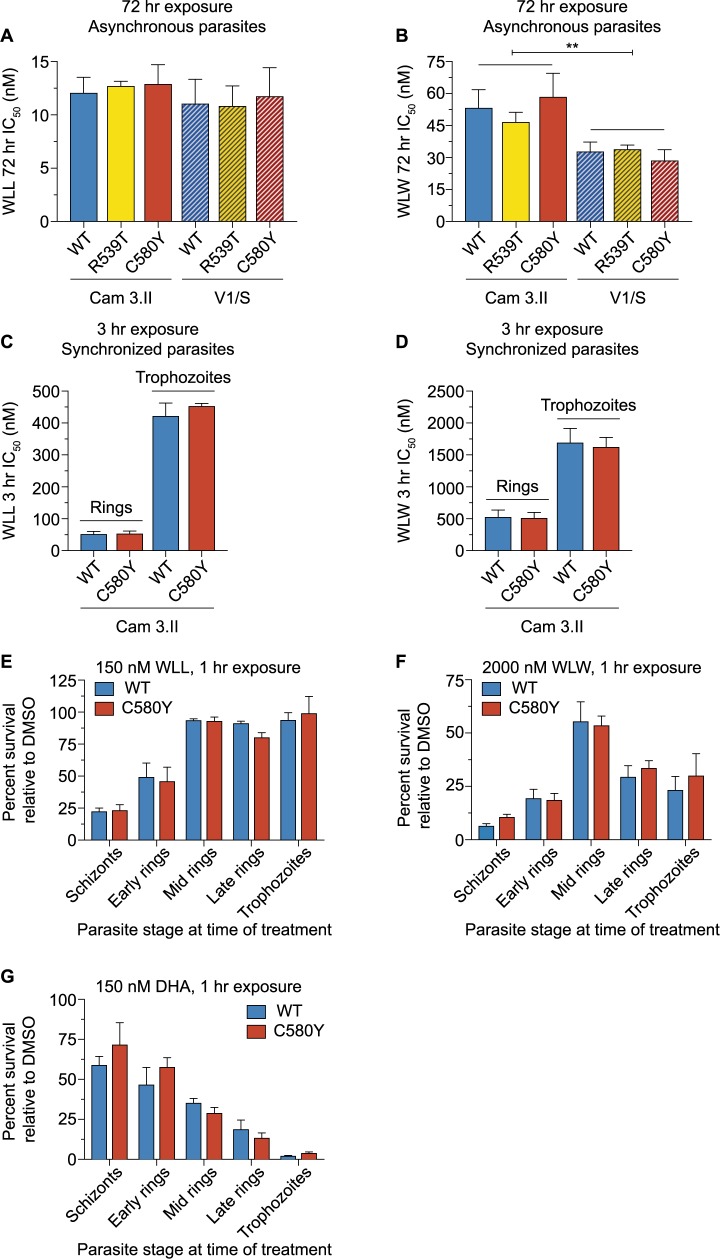
The *P*. *falciparum*-selective peptide vinyl sulfone proteasome inhibitors WLL and WLW are highly potent against schizonts and early ring-stage parasites irrespective of their K13 status. (A and B) Mean ± SEM IC_50_ values for (A) WLL or (B) WLW, tested in 72 hr dose-response assays with asynchronous parasites. These inhibitors were assayed against Cam3.II or V1/S parasites expressing either WT K13 or the ART-resistant R539T or C580Y variants. Assays were conducted on three to nine independent occasions in duplicate. Mann-Whitney *U* tests showed no significant differences between K13 WT lines and their isogenic variants on either background. IC_50_ values and statistics are reported in **S2 Table**. (C and D) Mean ± SEM IC_50_ values for (C) WLL or (D) WLW, tested in 3 hr exposures followed by washes to remove drug and a further 69 hr of culture in drug-free medium. Parasitemias were measured by flow cytometry. Assays were initiated with either early rings (0–3 hr post-invasion) or trophozoites (tested 24 hr after early rings). Assays were performed with Cam3.II or V1/S lines expressing K13 WT or C580Y, on three to four independent occasions in duplicate. Mann-Whitney *U* tests showed no significant differences between K13 WT lines and their isogenic variants. Two-way ANOVA tests comparing Cam3.II and V1/S parasite lines revealed no significant differences for WLL, and a small but significant difference for WLW. **P value = 0.005. IC_50_ values and statistics are reported in **S3 Table**. (E-G) Tightly synchronized Cam3.II parasites expressing K13 WT or C580Y were treated for 1 hr with (E) 150 nM WLL, (F) 2000 nM WLW, or (G) 150 nM DHA at five intervals, beginning with distinct stages. Drug was removed by washing and cultures were continued for a further 71 hr in drug-free medium prior to measuring parasitemias. Data are shown as mean ± SEM percent survival for drug-treated parasites, calculated relative to DMSO vehicle-treated cultures tested in parallel. Assays were performed on three (WLL and WLW) or two (DHA) independent occasions in duplicate. Percent survival values are reported in **S4 Table**.

Dose-response 72 hr assays with these six lines, tested as asynchronous cultures, revealed mean half-maximal inhibitory concentrations (IC_50_ values) in the range of 11–13 nM for the β2+β5 inhibitor WLL and 29–59 nM for the β2 inhibitor WLW (**Fig 1A and 1B** and **S2 Table**). When compared with isogenic WT K13 parasites, neither the C580Y nor the R539T variants displayed altered susceptibility to either proteasome inhibitor. V1/S parasites yielded slightly lower IC_50_ values than the Cam3.II lines in response to both inhibitors. For WLL, these differences were not statistically significant. However, for WLW, Cam3.II^C580Y^ showed a modest but nonetheless significant increase in IC_50_ as compared to V1/S^C580Y^. Two-way ANOVA showed a statistically significant, albeit small, difference in Cam3.II and V1/S IC_50_ values overall for WLW.

We also tested WLL and WLW against tightly synchronized early rings (0–3 hr post-invasion) to determine whether K13 mutations might alter ring-stage parasite susceptibility to *Plasmodium*-selective proteasome inhibitors. These experiments focused on the Cam3.II K13^WT^ and Cam3.II K13^C580Y^ isogenic pair. Parasites were exposed to a 3 hr pulse of WLL or WLW across a range of concentrations, after which the inhibitor was removed by repeated rounds of washing (see **Materials and Methods**). Cultures were continued for an additional 69 hr. These assays revealed IC_50_ values in the range of 52–54 nM for WLL and 516–531 nM for WLW for early rings, irrespective of their *K13* genotype. We also performed 3 hr pulse assays with synchronized Cam3.II K13^WT^ and Cam3.II K13^C580Y^ trophozoites, sampled 24 hr later, which yielded substantially higher IC_50_ values of 422–453 nM for WLL and 1628–1698 nM for WLW. With both rings and trophozoites, we saw no difference between isogenic Cam3.II lines expressing WT or mutant K13. Similar to the 72 hr assay, WLL was notably more potent than WLW at both stages (**Fig 1C and 1D** and **S3 Table**).

### WLL and WLW are potent inhibitors of *P*. *falciparum* early ring and schizont stages

We next examined the sensitivity of *P*. *falciparum* to the proteasome inhibitors WLL and WLW across the intra-erythrocytic developmental cycle by exposing tightly synchronized parasites to 1 hr drug pulses at specific intervals (45–47, 0–3, 10–13, 18–21 and 24–27 hr post-invasion). These experiments were conducted on the Cam3.II K13^WT^ and Cam3.II K13^C580Y^ isogenic pair. Parasites were exposed to fixed drug concentrations (150 nM for WLL and 2000 nM for WLW) for 1 hr, after which the inhibitor was removed by washout (see **Materials and Methods**). Cultures were then continued until 72 hr from the start of the experiment. Percent survival was calculated relative to mock (DMSO)-treated cultures. As an additional control, we also treated parasites with DHA at 150 nM.

In these 1 hr pulse assays, mature schizonts (treated just prior to or at the time of egress) and very early post-invasion rings were the most susceptible to proteasome inhibition by WLL and WLW (**Fig 1E and 1F**). Mid to late rings and trophozoites were less susceptible, exhibiting ~five-fold higher survival rates as compared to schizonts upon exposure to either inhibitor (**Fig 1E and 1F** and **S4 Table**). No differences were observed in the sensitivity profiles of Cam3.II K13^WT^ and Cam3.II K13^C580Y^ in response to either compound.

By comparison, DHA showed a dissimilar stage-specificity profile, with maximal potency against late rings and trophozoites. This profile coincides with the peak period of hemoglobin uptake and degradation, during which Fe^2+^-heme is liberated and activates ART [27,28]. Considerable inhibition was nonetheless observed in other stages, including early rings, which are also thought to undergo some digestion of hemoglobin to activate ART [29] (**Fig 1G**). Against schizonts, DHA treatment yielded almost twenty-fold higher survival levels than were observed with trophozoites (**S4 Table**). In these experiments, we observed only a minor increase in survival with K13 C580Y mutant parasites as compared to the isogenic K13 WT in early rings in response to a 1 hr pulse of 150 nM DHA, with no difference observed at later rings and trophozoites. Prior work has established that increasing the concentration (to 700 nM) and length of exposure (to 6 hr), in accordance with the ring-stage survival assay (RSA_0-3h_), leads to a significant gain in the survival rate of the K13 C580Y mutant, specifically in early rings [30–32].

To test the efficacy of our washout protocol, we also exposed uninfected red blood cells (RBCs) to the same 1 hr drug pulses described above (150 nM WLL, 2000 nM WLW, 150 nM DHA, or DMSO vehicle control). Uninfected RBCs were washed as per the 1 hr exposure assays described above. Magnet-purified, synchronized late trophozoites were then added to drug-treated RBCs or control (DMSO)-treated RBCs, and parasites were cultured for 48 hr to allow for one cycle of RBC invasion and parasite growth. Parasitemias were measured by flow cytometry and percent growth was calculated relative to DMSO control pre-treated wells. These assays were conducted with the Cam3.II K13^WT^ and Cam3.II K13^C580Y^ lines. Results showed that trophozoites added to WLL- or DHA- treated RBCs expanded to levels comparable to those added to DMSO-treated RBCs (96.0% and 99.8%, respectively, when averaged across K13 WT and K13 C580Y lines and across three independent experiments; **S5 Table**). These data show that WLL and DHA were both effectively washed out to sub-inhibitory concentrations (**Fig 1E and 1G**). By comparison, trophozoites inoculated into WLW-treated RBCs had reduced growth (79.7% on average across both lines relative to the DMSO control; **S5 Table**), which suggests that some of the inhibition observed in parasites treated with the high-dose of WLW (**Fig 1F**) may be attributable to drug carryover. Nonetheless, the inhibition profile observed for both WLL and WLW across stages was consistent, with both inhibitors showing maximal inhibition in schizonts and early ring stages, contrasting with maximum survival at the mid to late ring stage (**Fig 1E and 1F** and **S4 Table**).

### Parasites show a limited propensity for acquiring resistance to WLL or WLW

To evaluate the ability of *P*. *falciparum* to generate resistance to the proteasome inhibitors WLL and WLW, we exposed cultured parasites to sub-lethal concentrations of WLL or WLW (at either three or five times the IC_50_ level) for a period of up to 60 days. Selection studies were performed with the two pairs of isogenic K13 WT and C580Y lines: Cam3.II K13^WT^ and Cam3.II K13^C580Y^, and V1/S K13^WT^ and V1/S K13^C580Y^. Selections were performed in triplicate, each with a large starting inoculum of 2×10^9^ parasites per flask. Parasite clearance was confirmed during the first six days of treatment and subsequent recrudescence of parasitemia was monitored by microscopy two to three times a week.

Initial studies using 5×IC_50_ drug pressure for WLL showed very low levels of recrudescence, with only 2 of 12 selection flasks resulting in detection of parasites by day 60 (**Table 1**). Survival in the presence of WLW occurred more readily, with 6 of 12 flasks yielding recrudescent parasites. In the case of WLL, recrudescent parasites did not appear until day 51, whereas with WLW the mean time to recrudescence was 34 days for Cam3.II parasites and 27 days for V1/S parasites. A second round of selections performed under 3×IC_50_ drug pressure resulted in greater levels of recrudescence, with 6 of 12 WLL flasks and 12 of 12 WLW flasks yielding parasites within 24–41 days (**Table 1**). These results also suggested a lower propensity for the Cambodian Cam3.II line to develop resistance compared with V1/S (9 of 24 selections yielded parasites compared with 17 of 24, respectively). These data are consistent with earlier findings that V1/S was ~two-fold more mutable than Cam3.II [33] (Cam3.II was referred to therein as PH0306-C). Of note, K13 C580Y parasites were twice as likely to have positive selection outcomes when compared with K13 WT parasites (17 of 24 selections compared with 9 of 24 separate selections, respectively; **Table 1**), suggesting that the K13 C580Y mutation might also modestly increase the mutation rate.

**Table 1:** *In vitro* resistance selections.

Parasite line	Compound	Selection pressure^a^	Positive flasks^b^	Day first positive^c^
Cam3.II K13^WT^	WLL	5×IC_50_	0 of 3	—
Cam3.II K13^C580Y^	WLL	5×IC_50_	0 of 3	—
V1/S K13^WT^	WLL	5×IC_50_	0 of 3	—
V1/S K13^C580Y^	WLL	5×IC_50_	2 of 3	51, 51
Cam3.II K13^WT^	WLL	3×IC_50_	0 of 3	—
Cam3.II K13^C580Y^	WLL	3×IC_50_	2 of 3	35, 35
V1/S K13^WT^	WLL	3×IC_50_	1 of 3	41
V1/S K13^C580Y^	WLL	3×IC_50_	3 of 3	32, 38, 38
Cam3.II K13^WT^	WLW	5×IC_50_	0 of 3	—
Cam3.II K13^C580Y^	WLW	5×IC_50_	1 of 3	33
V1/S K13^WT^	WLW	5×IC_50_	2 of 3	27, 27
V1/S K13^C580Y^	WLW	5×IC_50_	3 of 3	27, 27, 27
Cam3.II K13^WT^	WLW	3×IC_50_	3 of 3	34, 34, 34
Cam3.II K13^C580Y^	WLW	3×IC_50_	3 of 3	34, 34, 34
V1/S K13^WT^	WLW	3×IC_50_	3 of 3	24, 24, 26
V1/S K13^C580Y^	WLW	3×IC_50_	3 of 3	29, 29, 31

^a^IC_50_ values for WLL and WLW are reported in S2 Table.

^b^Selections were performed with triplicate flasks each harboring an initial inoculum of 2×10^9^ asexual blood stage parasites.

^c^Cultures were stopped at Day 60 in the absence of recrudescence, indicating that no resistance was obtained.

### Whole-genome sequencing of WLL- or WLW-pressured parasites identifies point mutations in proteasome subunits

To identify genetic changes mediating parasite recrudescence following WLL and WLW selection, we performed Illumina-based whole-genome sequencing on the drug-pressured recrudescent lines and their four parental counterparts. In total, ten unique mutations were identified from the 26 recrudescent lines (**Table 2** and **S6 Table**). Four of the eight WLL-selected parasite lines harbored a mutation in the β5 subunit of the 20S proteasome core particle, resulting in an alanine to serine substitution at amino acid position 20 (A20S) in the mature protein, while the other four harbored mutations in the 20S proteasome β6 subunit, yielding either an alanine to valine change at position 117 (A117V) or a serine to leucine substitution at position 208 (S208L) (**Table 2** and **S6 Table**). 14 of 18 (78%) of WLW-selected parasite lines harbored a mutation in the 20S proteasome β2 subunit. These changes included a cysteine to phenylalanine or a cysteine to tyrosine mutation at position 31 (C31F or C31Y), and an alanine to glutamic acid substitution at position 49 (A49E). The four remaining lines selected against WLW harbored mutations in the 19S regulatory particle of the 26S proteasome. These mutations included a premature stop codon in RPT4 (E380*), two non-synonymous mutations in RPT5 (R295S and G319S), and a non-synonymous mutation in RPN6 (E266K) (**Table 2** and **S6 Table**).

**Table 2:** Mutations identified by whole-genome sequencing of WLL- or WLW-pressured parasite lines.

Gene ID	Protein name	Mutation pre-processing^a^	Mutation in mature protein^b^	Selection compound	Frequency^c^
PF3D7_1011400	20S β5 subunit	A80S	A20S	WLL	4/8
PF3D7_0518300	20S β6 subunit	A117V	A117V	WLL	3/8
PF3D7_0518300	20S β6 subunit	S208L	S208L	WLL	1/8
PF3D7_1328100	20S β2 subunit	C72F	C31F	WLW	7/18
PF3D7_1328100	20S β2 subunit	C72Y	C31Y	WLW	6/18
PF3D7_1328100	20S β2 subunit	A90E	A49E	WLW	2/18
PF3D7_1306400	19S RPT4	NA	E380*	WLW	2/18
PF3D7_1130400	19S RPT5	NA	G319S	WLW	1/18
PF3D7_1130400	19S RPT5	NA	R295S	WLW	1/18
PF3D7_1402300	19S RPN6	NA	E266K	WLW	2/18

^a^Amino acid change in protein prior to proteolytic processing.

^b^Amino acid change in mature protein (post-processing).

^c^Fraction of total drug-selected lines that acquired the designated mutation.

NA, not applicable (protein does not undergo post-translational processing).

In the 26 sequenced drug-pressured lines, we observed no other mutations at ≥20% allele frequency in the core genome (which removes sub-telomeric regions and members of multigene family members [34]). This observation further supports a primary role for the 19S and 20S proteasome mutations described above in mediating resistance. Copy number variation (CNV) analysis of the 26 lines detected amplification of a putative ubiquitin regulatory protein (PF3D7_0808300) in two recrudescent lines. In both cases, the amplification occurred in V1/S K13^C580Y^ parasites selected at 3×IC_50_ drug pressure with either WLL or WLW (**S6 Table;** flask R2 in both cases), suggesting that this gene may contribute to low-grade resistance to WLL and WLW. Both drug-pressured lines also harbored single nucleotide polymorphisms in the 20S proteasome β5 (A20S) or β2 (A49E) subunits, providing evidence that the ability of parasites to withstand drug pressure in the bulk cultures can be multifactorial. As a quality control for our CNV analysis, we confirmed that the V1/S and Cam3.II genomes differed at the GTP cyclohydrolase locus (PF3D7_1224000) that is amplified in V1/S parasites (contributing to high-grade pyrimethamine resistance; [35,36]). We also observed that V1/S parasites harbor a chromosome nine deletion that was previously associated with loss of cytoadhesion [37]. For these lines, the mean genome coverage was 54-fold (range 16–85; **S6 Table**).

### WLL- or WLW-selected lines display small IC_50_ increases and no cross-resistance between inhibitors

**Fig 2:**
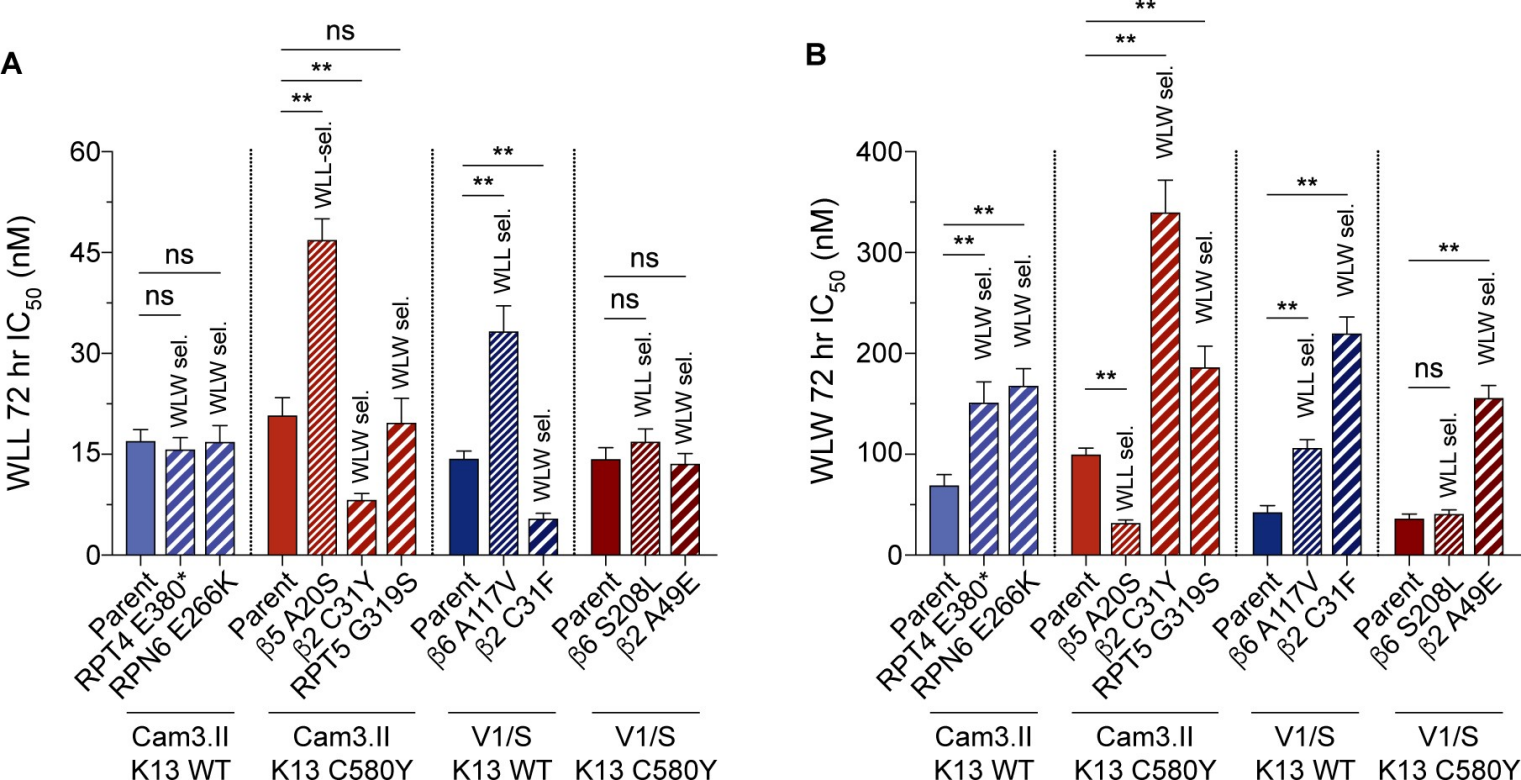
WLL- and WLW-pressured mutant parasite lines display modest gains of resistance. (A and B) Mean ± SEM IC_50_ values are shown for the Cam3.II or V1/S lines expressing (blue) WT or (red) C580Y K13, exposed to (A) WLL or (B) WLW for 72 hr. Assays were performed on five to seven independent occasions in duplicate. Shown above each bar is the compound used for resistance selections (thin hatching, WLL-selected; thick hatching, WLW-selected). Mutant lines (see **Table 2** and **S7 Table**) are illustrated beside their parental drug-sensitive line. Statistical significance was examined using Mann-Whitney *U* tests. **P value <0.01; ns, not significant. IC_50_ and IC_90_ values and associated statistics are reported in **S8 Table** and **S9 Table**, respectively.

From the set of WLL- or WLW-selected lines, we selected nine for 72 hr dose-response assays. Lines that contained a given proteasome mutation at ≥ 90% allele frequency were assayed directly without cloning, whereas lines that displayed mixed parasite populations were first cloned by limiting dilution. These lines are referred to herein by their unique proteasome mutations and are grouped by their respective parent: RPT4 E380* and RPN6 E266K (selected in the Cam3.II K13^WT^ parental line); β5 A20S, β2 C31Y and RPT5 G319S (Cam3.II K13^C580Y^); β6 A117V and β2 C31F (V1/S K13^WT^); and β6 S208L and β2 A49E (V1/S K13^C580Y^; **S7 Table**). In parallel, we also assayed the four parental lines: Cam3.II K13^WT^, Cam3.II K13^C580Y^, V1/S K13^WT^, and V1/S K13^C580Y^. Results are shown in **Fig 2**, in which K13 WT and K13 C580Y lines are colored blue and red, respectively. WLL-selected and WLW-selected lines are shown with thin and thick hatching, respectively.

WLL-selected lines harboring β5 A20S or β6 A117V mutations were observed to confer small (~two-fold) but nonetheless statistically significant increases in WLL IC_50_ levels compared with their parental lines (**Fig 2A** and **S8 Table**), and slightly higher increases (up to three-fold) in the WLL 90% inhibitory concentration (IC_90_ value; **S9 Table**). The WLL-selected β6 S208L line showed only very modest (<two-fold) and not statistically significant increases in WLL IC_50_ and IC_90_ values (**Fig 2A** and **S8 Table** and **S9 Table**). By comparison, WLW-selected lines harboring a β2 subunit mutation (C31Y, C31F and A49E) revealed slightly larger shifts (~three- to five-fold) in their WLW IC_50_ values compared to their parental lines (**Fig 2B** and **S8 Table**). WLW-selected lines harboring mutations in the 19S proteasome regulatory particle (RPT4 E380*, RPN6 E266K, and RPT5 G319S) displayed small (~two-fold) but significant increases in IC_50_ values compared with their parental lines. These trends were maintained at the IC_90_ level (**S9 Table**).

We next evaluated the ability of mutations identified in WLW-selected lines to confer resistance to WLL and vice versa. Surprisingly, we observed that the β2 C31F and β2 C31Y lines (both WLW-selected) were hypersensitive to inhibition by WLL, despite the fact that WLL inhibits both the β2 and β5 subunits of the parasite proteasome. The third β2 mutant line, β2 A49E, showed no shift in WLL IC_50_ or IC_90_ as compared to the parental line. Similarly, none of the 19S mutant lines displayed any cross-resistance to WLL (**Fig 2A** and **S8 Table** and **S9 Table**). The three mutations identified in WLL-pressured lines affected sensitivity to WLW in distinct ways: whereas the β5 A20S line showed significant hypersensitization to WLW, the β6 A117V line showed a minor (2.5-fold) increase in WLW IC_50_ as compared to the parental line. Finally, the β6 S208L mutation did not result in any significant shift in WLW IC_50_ or IC_90_ values (**Fig 2B** and **S8 Table** and **S9 Table**).

### Structure-based modeling suggests that mutations induce conformational rearrangements in the β2, β5 and β6 subunits

**Fig 3:**
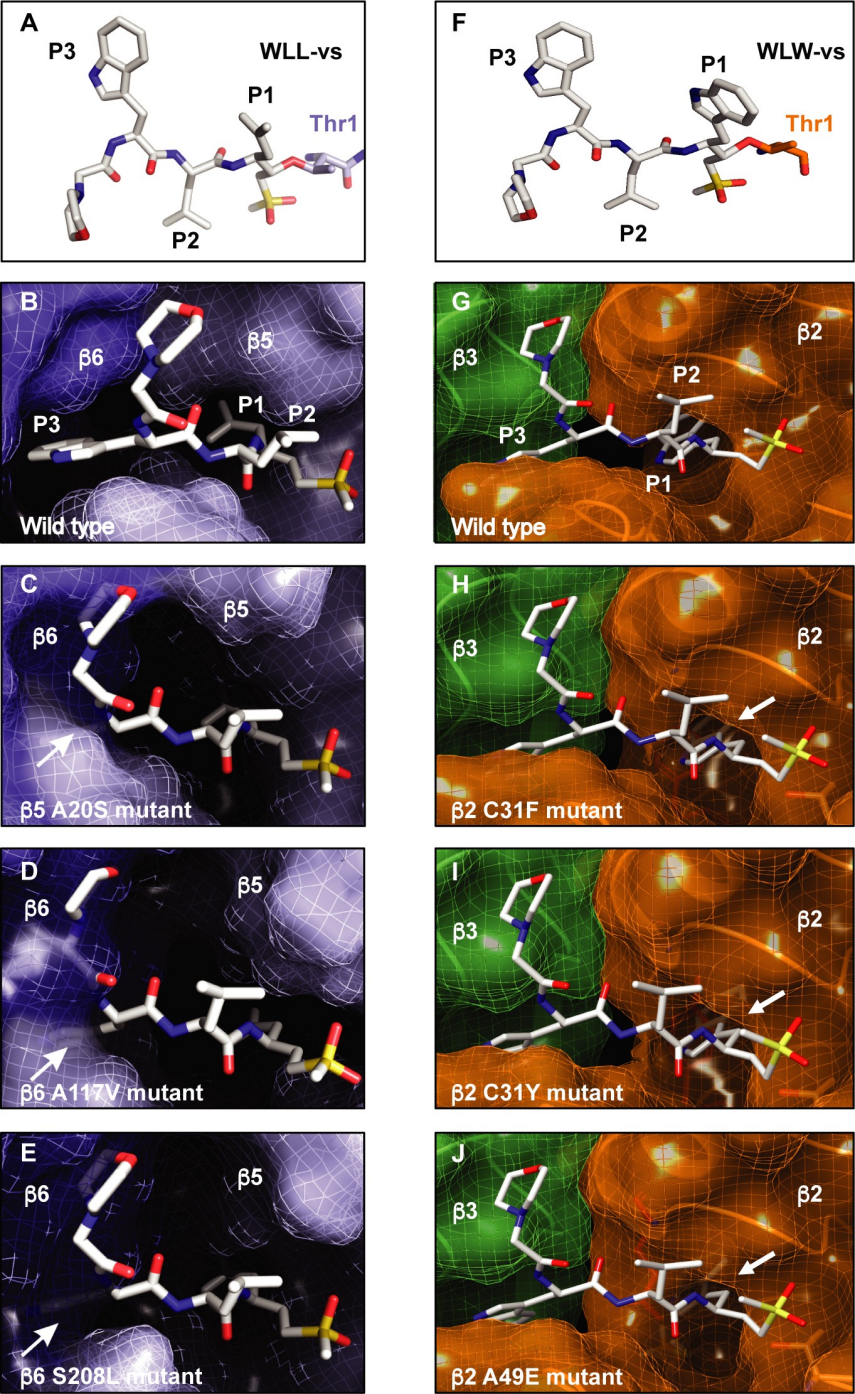
In silico modeling of 20S β2, β5 and β6 mutations reveals disruption of inhibitor binding. (A-E) Modeling of WLL binding. (A) Structure of the WLL peptide-based inhibitor, indicating positions P1-P3 and the electrophilic vinyl sulfone warhead that reacts with the β5 subunit catalytic threonine residue (Thr1). (B-E) Docking of WLL into the β5 active site. The β5 and β6 subunits are shown in light blue and dark blue, respectively. Arrows highlight the P3 tryptophan residue of the ligand. (B) Docking of WLL into the WT β5 site of the cryo-EM derived *P*. *falciparum* 20S proteasome model. (C-E) Docking of WLL into the β5 active site of molecular dynamics-simulated models with the WLL-selected mutations, namely (C) β5 A20S (D) β6 A117V and (E) β6 S208L, demonstrating their potential impact on WLL binding. (F-J) Modeling of WLW binding. (F) Structure of the WLW peptide-based inhibitor, indicating positions P1-P3 and the electrophilic vinyl sulfone warhead that reacts with the β2 subunit catalytic threonine residue (Thr1). (G-J) Docking of WLW into the β2 active site. The β2 and β3 subunits are shown in orange and green, respectively. Arrows highlight the P1 tryptophan residue of the ligand. (G) Docking of WLW into the WT β2 site of the cryo-EM derived *P*. *falciparum* 20S proteasome model. (H-J) Docking of WLW into the β2 active site of the superposed WLW-selected mutation models, i.e. (H) β2 C31F (I) β2 C31Y and (J) β2 A49E, and their potential impact on WLW binding.

To simulate the effects of mutations in the β2, β5 and β6 subunits on WLL and WLW binding *in silico*, we performed structural analyses with both inhibitors using the high resolution cryo-EM based structure of the *P*. *falciparum* 20S proteasome (PDB accession code 5FMG). For these studies, we docked WLL into the β5 active site of the cryo-EM-based atomic model of the *P*. *falciparum* 20S proteasome (**Fig 3A** and **3B**) and for WLW used the previously solved structure of the inhibitor bound to the β2 active site (**Fig 3F** and **3G**) [21]. As expected, docking studies revealed that WLL was well-accommodated within the β5 active site (**Fig 3B**).

To examine the effect of the WLL-selected mutations β5 A20S and β6 A117V and S208L, we used molecular dynamics simulations to individually evaluate the effects of these mutations on the β5 active site (**Fig 3C–3E**). For the β5 A20S mutation, the introduction of a serine side chain was predicted to directly impose steric constraints on the S3 binding pocket of the β5 active site. These constraints would not favor large groups at the P3 position of the ligand, such as the P3 tryptophan of WLL. The β6 A117V mutation was predicted to destabilize a cluster formed by three tyrosine residues at positions 150, 152 and 158 of β6, inducing a slight displacement of the β strands of the β6 subunit towards the β5 binding pocket. This conformational change would produce steric constraints on the β5 active site binding pocket, particularly for access of the WLL P3 group, as recently suggested for the similar β6 A117D substitution [23]. For the β6 S208L mutation, introduction of the leucine side chain in β6 was predicted to cause a significant clash with the adjacent α helix in the β3 subunit. This substitution induced conformational rearrangements in the model that were able to propagate as far as the S3 site of the β5 binding pocket. These changes would again impose steric constraints on WLL binding (**Fig 3C–3E**). The rearrangements induced by the WLL-selected mutations were all characterized by smaller, sterically-constrained β5 binding pockets, particularly at the S3 position, as compared with the WT *Plasmodium* proteasome β5 active site.

We also modeled the WLW-selected β2 mutations, namely C31Y, C31F and A49E, using the known cryo-EM based *P*. *falciparum* 20S proteasome structure (5FMG; [21]). The three mutated residues are located near the S1 binding pocket of the β2 active site. The introduction of bulky tyrosine or phenylalanine residues in place of the cysteine at position 31 was predicted to cause a steric clash with the large P1 tryptophan of WLW, likely preventing binding of WLW to the β2 subunit (**Fig 3H** and **3I**). Similarly, the β2 A49E mutation was predicted to produce steric constraints near the S1 binding pocket caused by the introduction of the bulky glutamate side chain (**Fig 3J**). Structural modeling suggested that these β2 mutants should still retain sensitivity to WLL, which has a smaller P1 side chain compared to WLW. These predictions are consistent with the lack of cross-resistance between the WLW-selected mutants and WLL; in fact, the β2 C31Y and C31F mutant lines selected under WLW pressure showed hypersensitivity to WLL (**Fig 2**).

### Activity-based probe labeling reveals inhibitor interactions with proteasome subunits that are affected by mutations in drug-pressured lines

We next examined whether mutations in the 20S proteasome subunits confer resistance to WLL or WLW by directly precluding binding of the inhibitors to the active sites of the proteasome. These experiments involved activity-based probe (ABP) labeling of the three catalytic subunits β1, β2 and β5 using the proteasome active-site fluorogenic probe BMV037 ([26,38,39]; see **Materials and Methods**). This probe competes for binding with proteasome-specific inhibitors including WLL and WLW, which allows for direct assessment of inhibitor binding to each of the active beta subunits of the proteasome through the quantification of residual protein labeling after inhibitor treatment.

**Fig 4:**
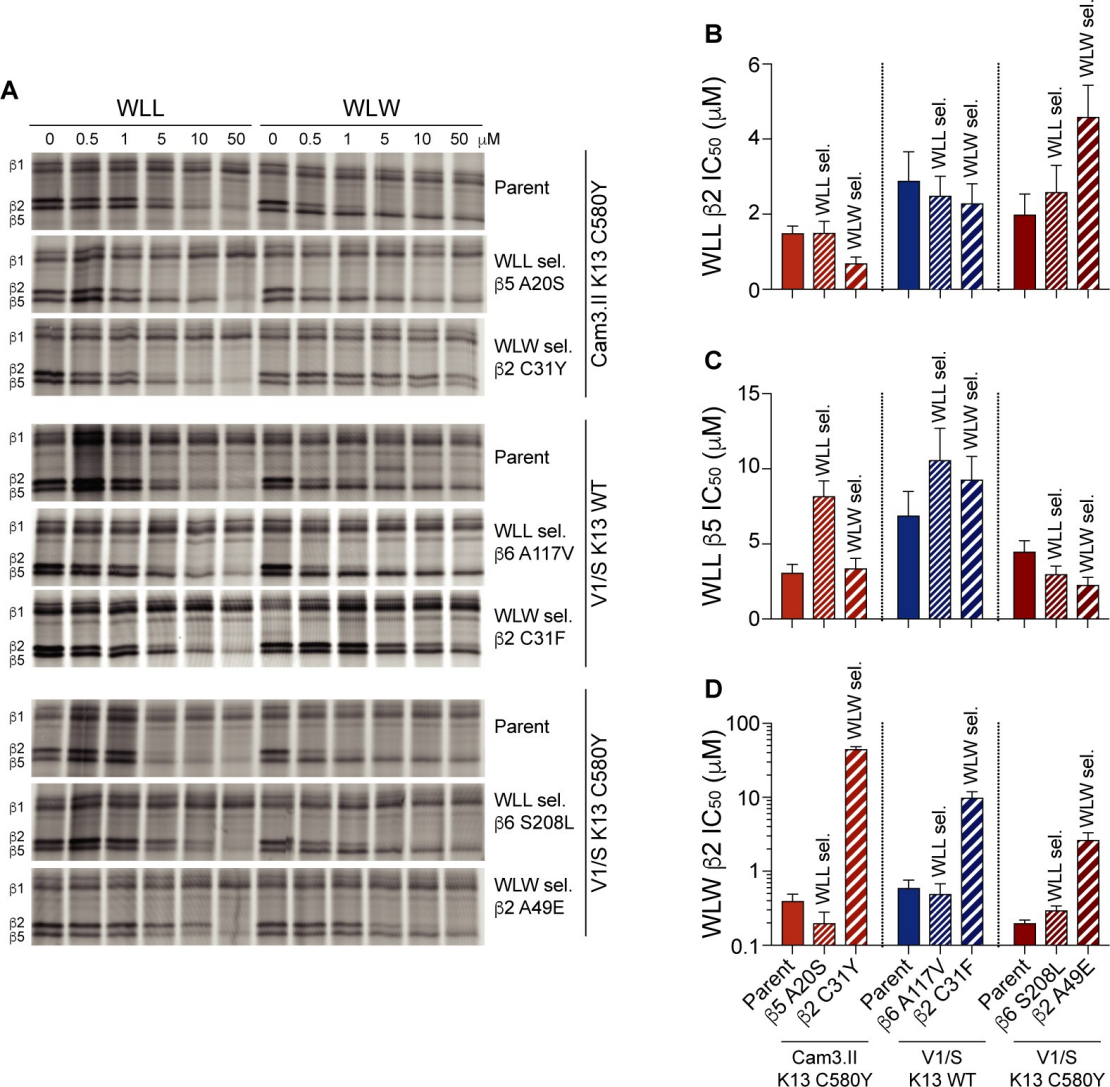
Activity-based probe profiling of 20S β2 and β5 active sites permits quantification of the impact of 20S subunit mutations on inhibitor binding. (A) *P*. *falciparum* schizont lysates treated for 1 hr with WLL or WLW at concentrations ranging from 0.5 to 50 μM, or mock-treated with DMSO, then incubated for 2 hr with the BMV037 probe. Samples were run on 12% SDS-PAGE gels. Data show results from one representative experiment. (B-D) Bar charts showing the concentrations at which WLL or WLW achieved half-maximal inhibition of probe labeling to each subunit. Data are shown as mean ± SEM IC_50_ values from two independent experiments (of which one is shown in panel A). (B) Inhibition of β2 subunit by WLL; (C) Inhibition of β5 subunit by WLL (D); inhibition of β2 subunit by WLW. IC_50_ values are reported in **S10 Table**.

Competition assays were performed for the three WLL-selected lines harboring β5 or β6 mutations (β5 A20S, β6 A117V, and β6 S208L) and the three WLW-selected lines harboring mutations in β2 (C31Y, C31F and A49E). *P*. *falciparum* schizont lysates from the six test lines and their respective parents were pre-incubated for 1 hr with WLL or WLW at concentrations ranging from 0.5 to 50 μM, or mock-treated, then incubated for 2 hr with the BMV037 probe. In the absence of either inhibitor, the probe showed similar labeling regardless of whether lines carried mutations in the proteasome subunits or not (**Fig 4A**). In the presence of increasing concentrations of WLL or WLW, probe labeling of the β2 and β5 subunits was reduced by the binding of these inhibitors. To quantify this effect, we calculated the concentration at which WLL or WLW achieved half-maximal inhibition of probe labeling to each subunit (shown as IC_50_ values in **Fig 4B–4D** and **S10 Table**; see **Materials and Methods**). For the WLL-selected lines, the β5 A20S mutation had a small effect on WLL binding to β5, manifesting as a slight increase in the WLL β5 IC_50_ (**Fig 4A** and **4C**). This result is consistent with our structural analysis. This mutation, however, did not impact inhibition of the β2 subunit by either WLL or WLW (**Fig 4A, 4B** and **4D**). The WLL-selected β6 mutation A117V slightly reduced the potency of WLL in blocking the labeling of the β5 subunit by BMV037, whereas the β6 S208L mutation appeared to have a minor effect on WLL binding to β2. These two β6 mutations did not substantially affect binding of WLW to the β2 active site (**Fig 4A–4D**).

For the WLW-selected lines, the β2 C31Y and C31F mutations both reduced WLW binding to the β2 active site, but did not prevent WLL binding to β2 (**Fig 4A–4D**). This finding agrees with our structural analysis, which shows a preference against mutant site occupancy by the large tryptophan P1 group of WLW. This also explains why the WLW-selected mutants were not resistant to WLL, which has a leucine in the P1 position. The third WLW-selected β2 mutation, A49E, also prevented WLW binding to β2, but not as strongly as C31Y or C31F. This mutation resulted in a reduction of WLL binding to β2, which is consistent with the proximity of this mutation to the entrance of the β2 binding pocket [40]; **Fig 4A–4D**).

### Proteasome inhibitors WLL and WLW synergize with multiple classes of antimalarial agents

**Fig 5:**
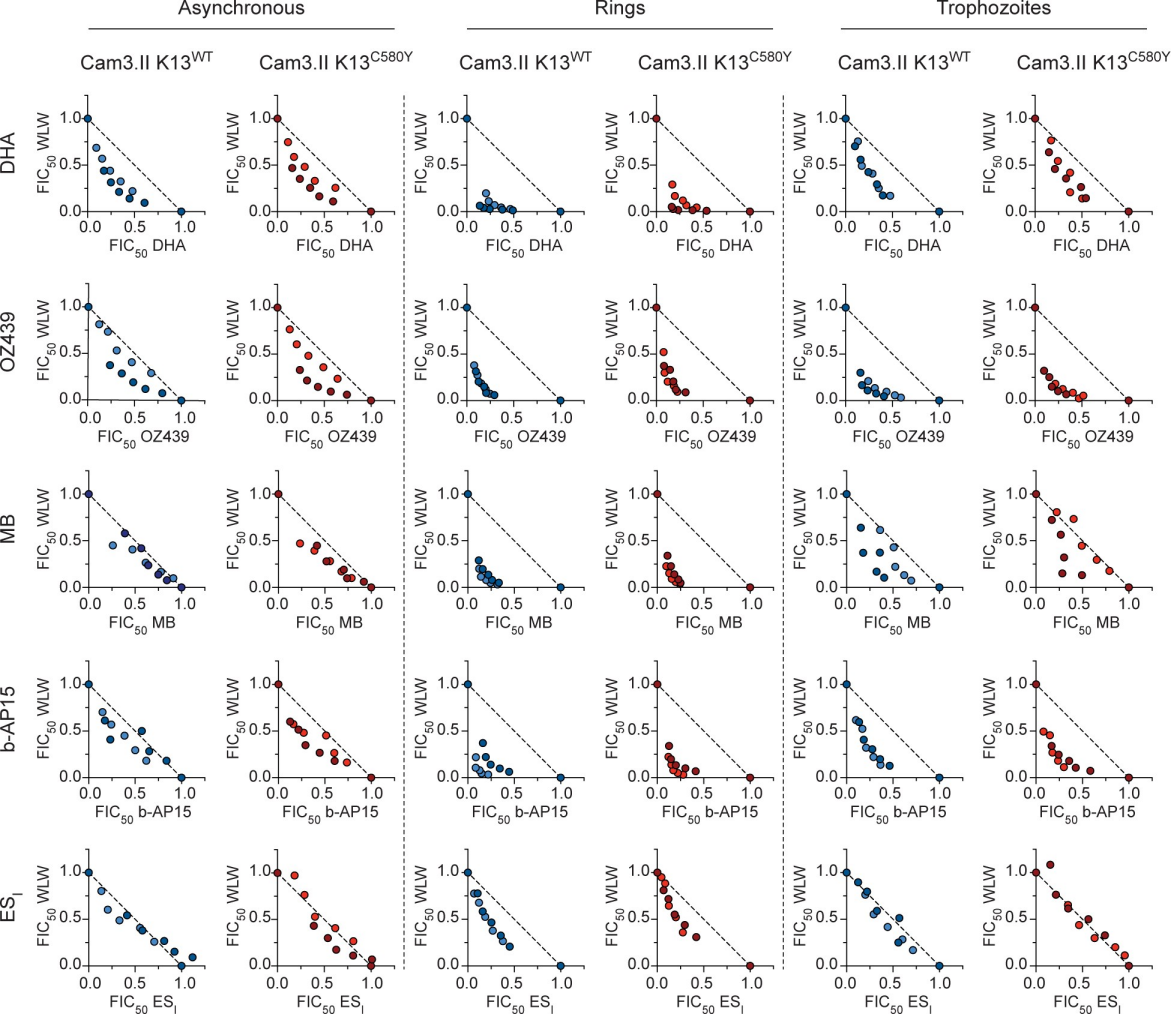
WLW synergizes with multiple classes of structurally and functionally diverse antimalarials. Isobolograms of WLW and (in descending order) DHA, OZ439, MB, b-AP15 and ES_I_ tested on asynchronous parasites and highly synchronized rings and trophozoites. Cam3.II K13^WT^ or Cam3.II K13^C580Y^ parasites were exposed to compounds mixed at fixed ratios of their individual IC_50_ values (1:0, 4:1, 2:1, 1:1, 1:2, 1:4, 0:1). Asynchronous parasites were exposed for 72 hr and parasitemias were determined by flow cytometry. Highly synchronized rings (0–3 hr post-invasion) or trophozoites (tested 24 hr later) were exposed for 3 hr, followed by drug washouts and continued culture for 69 hr in drug-free media. Fractional IC_50_ (FIC_50_) values were plotted for each drug combination and fixed ratio and results were compared against a hypothetical isobole line illustrating a perfectly additive interaction (dashed line). Data show results of two independent isobologram assays (shown in different shades), each performed in duplicate, tested against Cam3.II K13^WT^ (blue) and Cam3.II K13^C580Y^ (red). Synergy is evidenced by individual FIC_50_ pairwise values falling below the dashed line of additive interactions (FIC_50_ = 1). DHA, dihydroartemisinin; ES_I_, eeyarestatin I; MB, methylene blue.

To test for interactions between WLL or WLW and other classes of antimalarials, we performed isobologram assays with the isogenic ART-S Cam3.II K13^WT^ and ART-R Cam3.II K13^C580Y^ lines. These assays included DHA, the related endoperoxide-containing compound OZ439 that is suspected to have a similar mode of action [41,42], methylene blue (MB) that disrupts redox homeostasis, and two compounds implicated in pathways related to the UPS, namely b-AP15 that inhibits proteasome-associated deubiquitinases, and eeyarestatin I (ES_I_) that inhibits the ER-associated degradation (ERAD) pathway (**S11 Table**). Assays were initiated with either asynchronous parasites that were exposed to drugs for 72 hr, or with synchronized early rings (0–3 hr post-invasion) or trophozoites (tested 24 hr later) that were exposed to drugs for 3 hr followed by three rounds of drug washout and a further 69 hr of incubation in drug-free medium prior to measuring parasitemias. Drug combinations were tested at fixed ratios (1:0, 4:1, 2:1, 1:1, 1:2, 1:4, 0:1) across a range of concentrations (see **Materials and Methods**). From these data, we derived fractional IC_50_ (FIC_50_) values for the two compounds at each of the ratios tested, and plotted these values on isobologram graphs. The shapes of the resulting curves were then compared against a hypothetical isobole line illustrating a perfectly additive interaction (dashed line in **Fig 5**). With these graphs, a concave curve with points lying substantially below the isobole line is evidence of synergy, whereas points near the isobole line indicate additivity, and a convex curve with points lying substantially above the isobole line indicates antagonism.

Our drug combination studies provided clear evidence of synergy between WLW and DHA, OZ439, MB and b-AP15, for both the ART-S and ART-R isogenic lines (**Fig 5**). These results were obtained with asynchronous parasites, as well as synchronized early post-invasion rings and trophozoites. Rings showed the clearest evidence of synergy, as evidenced by the most concave curves. This finding was particularly significant as rings are generally the least susceptible to antimalarial drugs including ART derivatives (**Fig 1G**; [43]), with the notable exception of proteasome inhibitors that our data show are the most potent against this stage. Mild synergy was observed between WLW and ES_I_ at the early ring stage, however the interaction between these two compounds was additive on asynchronous parasites and on trophozoites (**Fig 5**). Synergy was also evidenced with DHA, OZ439 and b-AP15 in combination with WLL on early rings and trophozoites, though to a reduced degree as compared with WLW (**S1 Fig**). For MB, synergy with WLL was limited to the early ring stage, and for ES_I_, the interaction with WLL was largely additive (**S1 Fig**).

**Fig 6:**
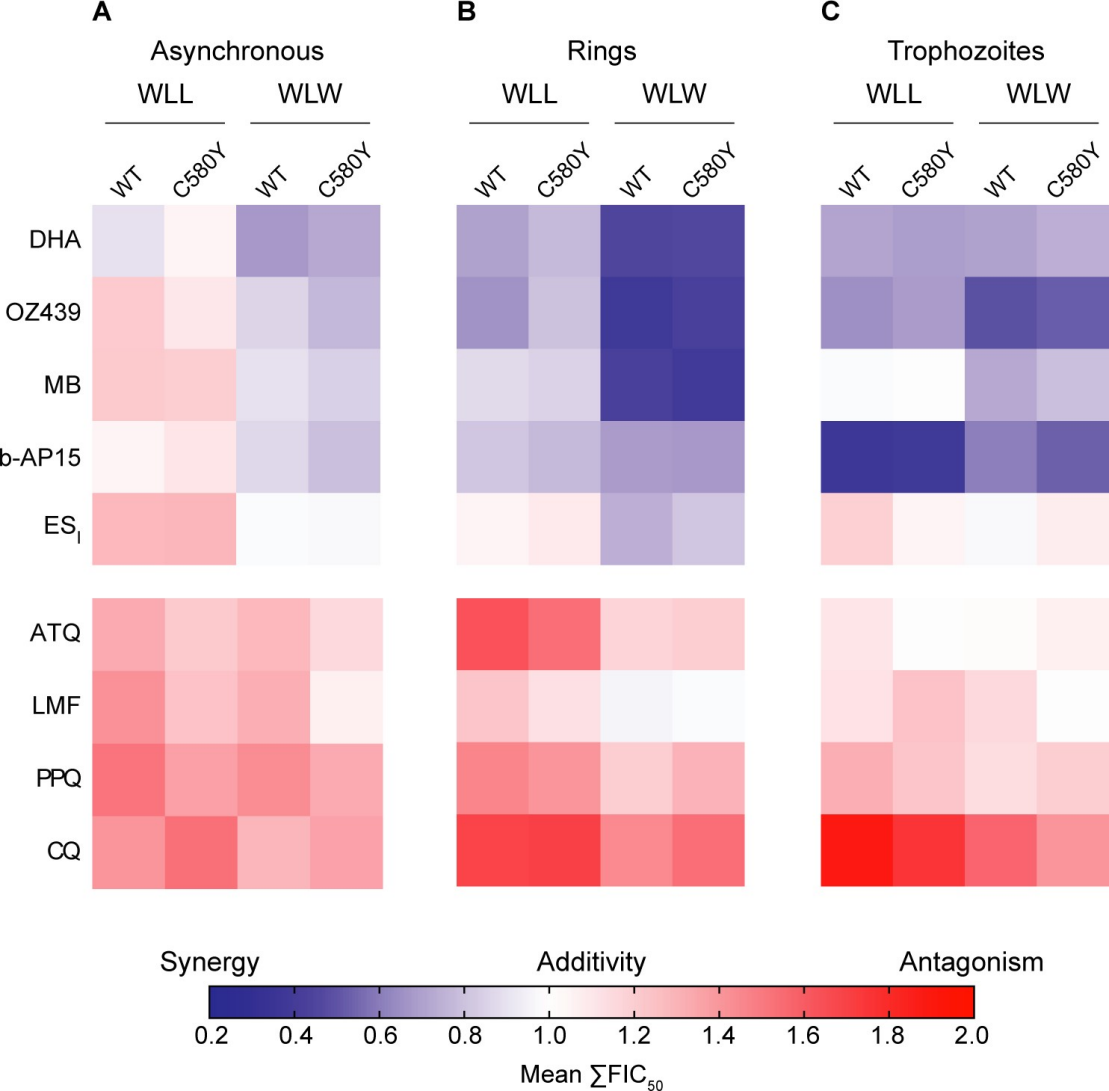
WLL and WLW show differential interactions with distinct classes of antimalarials, including synergy with DHA and the related ozonide OZ439, and antagonism with CQ and PPQ. (A-C) Heat maps of interactions between the WLL or WLW proteasome inhibitors and distinct antimalarial agents. Assays used the Cam3.II K13^WT^ and Cam3.II K13^C580Y^ lines. Parasites were exposed to compounds mixed at fixed ratios of their individual IC_50_ values (1:0, 4:1, 2:1, 1:1, 1:2, 1:4, 0:1). (A) Asynchronous parasites were exposed for 72 hr and parasitemias were determined by flow cytometry. (B) Highly synchronized rings (0–3 hr post-invasion) or (C) trophozoites (tested 24 hr later) were exposed for 3 hr, followed by drug washouts and continued culture for 69 hr in drug-free media. Values represent the mean of the sums of the FIC_50_ values over the five fixed ratios of the two test compounds (excluding the 1:0 and 0:1 points). Assays were conducted on two to four independent occasions in duplicate. Data for WLW and the five top compounds (DHA, OZ439, MB, b-AP15 and ES_I_) are presented as isobolograms in Fig 5. ATQ, atovaquone; CQ, chloroquine; DHA, dihydroartemisinin; ES_I_, eeyarestatin I; LMF, lumefantrine; MB, methylene blue; PPQ, piperaquine. Means of the sums of FIC_50_ (mean ΣFIC_50_) values are reported in **S12 Table**.

We extended our isobologram studies to the mitochondrial inhibitor atovaquone (ATQ), the licensed ACT partner drugs and suspected heme-interacting agents lumefantrine (LMF) and piperaquine (PPQ), and the former first-line antimalarial chloroquine (CQ; **S11 Table**). Additive to antagonistic profiles were observed between the proteasome inhibitors and these four compounds. Results for these compounds and those tested above are shown as the mean of the sums of the fractional IC_50_ values of the combinations (mean ΣFIC_50_) and are represented as heat maps (**Fig 6**; values tabulated in **S12 Table**). A mean value less than or equal to 0.5 indicates that the interaction between the two compounds was potently synergistic (blue), a mean close to 1.0 is indicative of an additive interaction (white), and a mean greater than or equal to 1.5 indicates potent antagonism (red). These thresholds are rarely met with *P*. *falciparum*, in part because such interactions manifest the most clearly only at certain combination ratios [21,44–46]. Mean ΣFIC_50_ values lying between these cutoffs, i.e. greater than 0.5 but less than 1.0, or greater than 1.0 but less than 1.5, suggest mild synergy or mild antagonism, respectively.

For LMF, mild antagonism was observed on asynchronous parasites with WLL or WLW, while these interactions were largely additive on early rings and trophozoites. For PPQ and ATQ, the antagonism observed in asynchronous parasites and early rings was attenuated in trophozoites. For CQ, moderate to potent antagonism was observed across all stages (**Fig 6A–6C**). These heat maps visually illustrate the potent synergy observed with DHA, OZ439, MB and b-AP15 (**Fig 5**).

We also tested a panel of experimental compounds with diverse targets and modes of action, including AN3661, an inhibitor of the *P*. *falciparum* cleavage and polyadenylation specificity factor subunit 3; ACT-451840, suspected to inhibit PfMDR1; cycloheximide, an inhibitor of tRNA translocation and protein synthesis; DDD107498, an inhibitor of *P*. *falciparum* translation elongation factor 2; DSM265, a dihydroorotate dehydrogenase inhibitor; halofuginone, a prolyl tRNA synthetase inhibitor; and NITD609, an inhibitor of *P*. *falciparum* P-type Na^+^ ATPase 4 (**S11 Table**) [47–53]. Each compound showed antagonism with WLL and WLW. No substantial differences were observed in the responses of K13 WT versus K13 C580Y parasites to any of these compounds (**S2 Fig** and **S13 Table**).

## Discussion

The evolution of drug-resistant *P*. *falciparum* parasites has severely compromised prior-generation first-line antimalarials such as CQ, with devastating consequences. Resistance is also increasingly undermining the efficacy of ACTs in SE Asia. The search for new antimalarials has uncovered a spectrum of novel scaffolds active against specific parasite targets, including mitochondrial factors (DHODH, cytochrome B), the Na^+^-ATPase PfATP4, the cis-Golgi protein PfCARL, several cytosolic tRNA synthetases, and the eukaryotic elongation factor PfeEF2 [54,55]. Nonetheless, despite their nanomolar potency, many of these inhibitors can readily select for resistance via mutations in their respective target proteins, sometimes with as few as 10^6^ or 10^7^ parasites. Also, depending on the target and inhibitor, the IC_50_ increases observed in resistant parasites can vary from several fold up to two thousand-fold [54]. These findings can have direct implications for treatment outcomes *in vivo*, as recently evidenced in a clinical trial where two of 24 *P*. *falciparum*-infected patients treated with a low single dose of DSM265 recrudesced with parasites that had acquired DHODH mutations previously observed in DSM265 resistance selections *in vitro* [56].

Here, we report that the *Plasmodium* proteasome-specific peptide vinyl sulfone inhibitors WLL and WLW have low nanomolar potency against genetically diverse parasites, are equally effective against parasites expressing mutant or WT forms of the ART resistance determinant K13, and display a unique stage-specificity profile. Stage-specificity assays reveal WLL and WLW to be most potent against schizonts and early ring stages. This finding is particularly promising as the majority of currently-employed antimalarials are most active against the trophozoite stage, including ART derivatives (which also inhibit rings) [43]. Importantly, we also observe a minimal resistance liability with WLL and WLW. Inocula of 2 × 10^9^ parasites exposed to low drug levels yielded recrudescent parasites in only half the selections, with IC_50_ increases averaging <three-fold (**Table 1** and **S8 Table**). WLL- or WLW-resistant lines retained full sensitivity to the alternate inhibitor, and in several cases mutations that conferred resistance to one inhibitor sensitized parasites to the other. This finding suggests that resistance is highly compound-specific and that selective pressures exerted by different inhibitors even within the same series can act in opposing directions. The different selectivity of WLL and WLW was confirmed by activity-based profiling of the proteasome catalytic sites in parasites harboring mutations in β2, β5 or β6, which revealed that WLL-selected mutations interfered specifically with binding of WLL and not WLW, and vice versa. We also leveraged the existing high-resolution cryo-EM structure of the *P*. *falciparum* 20S proteasome [40] to perform *in silico* modeling, which predicted that mutations in the β2, β5 and β6 subunits could specifically reconfigure proteasome active sites in the 20S core particle to confer compound-selective resistance. Gene-editing studies, which would provide an additional layer of confirmation, have not yet been undertaken.

Of note, a recent study of noncovalent, reversible asparagine ethylenediamine (AsnEDA) 20S proteasome inhibitors reported parasites that were cross-resistant to several AsnEDA inhibitors but were hypersensitized to the pan-active proteasome inhibitors bortezomib and carfilzomib, as well as to WLW [23]. This finding adds to the evidence that proteasome inhibitor resistance can be class-, and, in the case of the vinyl sulfones, even compound-specific. Resistance selections with the AsnEDA compound PKS21004 yielded parasites with high levels of resistance (>130-fold increases over the sensitive parent), and yielded mutations in the same residue as one of our WLL-pressure lines (β6 A117D for PKS21004 versus β6 A117V for WLL).

These differing levels of resistance may be attributable to distinct modes of inhibition for the AsnEDA compounds versus the vinyl sulfone inhibitors, notably the ability of the latter to form permanent covalent linkages with the active site threonine of the proteasome beta subunits. Vinyl sulfone-mediated inhibition is controlled by an initial reversible binding event and the subsequent formation of a covalent adduct with the proteasome active site. Mutations identified in our resistant lines may alter the initial binding of the inhibitor, which would in turn limit the rate of covalent modification of the active site. However, the potency of these inhibitors would only be nominally compromised because in the mutant enzyme covalent adducts would still accumulate over time. For reversible binding compounds such as the AsnEDA series, these same mutations are predicted to lead to reduced steady-state levels of inhibitor-bound active sites, causing higher resistance levels. Our data suggest that compounds with covalent modes of inhibition may thus be preferable over reversible binding inhibitors in helping to reduce the risk of high-grade resistance.

We also sought to explore potential partner agents for combination therapies including proteasome inhibitors. Results from isobologram studies revealed potent synergy between WLL or WLW and five distinct antimalarial agents: DHA, OZ439, methylene blue, b-AP15, and ES_I_. Synergy was the most pronounced with early rings, highlighting the value of assessing drug-drug interactions with synchronized cultures. Our data suggest that these structurally diverse compounds might share a common feature of generating damaged or misfolded proteins that accumulate as UPS substrates. Given that the UPS is a major regulator of the cell stress response, we propose that inhibition of the *P*. *falciparum* proteasome precludes parasites from resolving protein damage caused by these compounds, thereby creating synergy. DHA and OZ439, for instance, are endoperoxide-containing drugs that generate carbon-centered radical species, which non-specifically alkylate intracellular heme and other biomolecules in blood-stage parasites [41,42,57,58]. This activity is suspected to increase the burden of misfolded and damaged proteins. We confirmed earlier observations of proteasome inhibitor synergy with DHA [15,21–25]. Leveraging the availability of isogenic K13 WT and mutant parasites we also showed that synergy was unaffected by K13 sequence. Our studies also revealed potent synergy between WLL or WLW and OZ439, an ozonide compound related to ART that is now in human clinical trials [2,59,60].

MB is also of interest for potential combination therapies, as this redox-perturbing drug is very potent against *Plasmodium* asexual blood stages and gametocytes and has proven gametocytocidal efficacy in *P*. *falciparum*-infected patients [61–63]. Its activity has been attributed in part to disruption of glutathione (GSH) redox cycling [64]. GSH is an antioxidant that neutralizes cellular damage by reactive oxygen species (ROS). The major source of ROS in *P*. *falciparum* is parasite-mediated hemoglobin proteolysis and the oxidation of liberated, reactive heme. Destabilization of the parasite’s antioxidant defense mechanisms results in widespread damage to cell membranes, proteins and other molecules [65]. Similar to DHA and OZ439, treatment with MB might increase the parasite’s reliance on the UPS for clearance of damaged biomolecules, creating synergy with proteasome inhibitors. Prior experiments have also shown synergy between MB and ART derivatives, further indicating that both drugs may activate similar cellular defense pathways in response to protein damage, including the UPS [66,67].

Of the two experimental compounds that we tested, one, the anti-cancer agent b-AP15, acts directly on the UPS, inhibiting the activity of proteasome-associated deubiquitinases (DUBs) that catalyze the deubiquitination of proteasome-targeted substrates prior to their translocation into the 20S proteolytic machinery [68,69]. Interference with ubiquitin deconjugation from target substrates prevents polypeptide translocation into the 20S proteasome catalytic core and abrogates proteasomal degradation [17]. The antimalarial activity of b-AP15 has been attributed to inhibition of PfUSP14 and/or PfUCH54, two 19S-subunit associated DUBs [68–70]. The second compound, ES_I_, inhibits the UPS-related ER-associated degradation (ERAD) pathway that mediates the disposal of misfolded ER-resident or trafficked proteins [71,72]. In ERAD, misfolded proteins are shuttled out of the ER through a retrotranslocation machinery into the cytoplasm where they are ubiquitinated and subsequently degraded by the proteasome [72]. In human cancer cell lines, ES_I_ specifically inhibits p97, an AAA-ATPase that is an essential component of the ER retrotranslocon [73,74], suggesting this as a potential target in *Plasmodium*. Synergies observed between our proteasome inhibitors and both ES_I_ and b-AP15 can likely be attributed to the disruption of two targets in the UPS pathway. Both sets of interactions suggest promising avenues for developing novel combination therapies.

Our data also identified several antimalarial classes that were antagonistic with proteasome inhibitors. These included inhibitors of hemoglobin metabolism and heme detoxification (CQ, PPQ and putatively LMF), mitochondrial function (ATQ and DSM265), protein synthesis (AN3661, CHX, DDD107498 and HFG), sodium homeostasis (NITD609), and digestive vacuole transport processes (ACT-451840) [54,55]. These drugs may antagonize proteasome inhibitors by reducing the protein degradative burden on the UPS. As an example, attenuating protein synthesis through translation inhibitors (such as DDD10798 or CHX) could reduce the parasite’s reliance on the proteasome to eliminate defective nascent proteins and thereby diminish the impact of proteasome inhibition [16,75].

For the 4-aminoquinolines CQ and PPQ, antagonism of WLL and WLW might be attributable to their inhibition of hemoglobin proteolysis, which occurs at similar concentrations to their inhibition of hemozoin formation [76]. This may lead to translation attenuation due to a lack of available amino acid precursors stemming from liberated and digested globin, thereby reducing dependency on the proteasome and antagonizing its inhibitors. This postulate could be addressed by studying hemoglobinase inhibitors, such as ALLN and E-64 [45,77]. Further studies are required to determine whether inhibition of mitochondrial functions (specifically pyrimidine biosynthesis and maintenance of the electron transport chain, inhibited by DSM265 and atovaquone respectively) would also antagonize proteasomal inhibitors by attenuating protein synthesis.

Our findings that WLL and WLW share a low propensity for selecting for parasite resistance and favorable stage-specificity and synergy profiles provide a compelling case for the continued development of *Plasmodium*-selective proteasome inhibitors as antimalarial therapeutics. Ongoing efforts are focused on improving selectivity and pharmacological properties of the lead vinyl sulfone inhibitors. Recently, we reported a new set of optimized peptide vinyl sulfone inhibitors, chemically related to WLL, which retained their potency and synergy with DHA and displayed over three orders of magnitude selectivity for the *P*. *falciparum* enzyme. These compounds had improved solubility, metabolic stability, and oral bioavailability, and were active in a *P*. *berghei* rodent malaria model [24]. The data presented herein reveal multiple chemical classes that display synergistic interactions with peptide vinyl sulfones, and highlight covalent proteasome inhibitors as promising new agents for use in resistance-refractory combination therapies to treat multidrug-resistant malaria.

## Materials and methods

### Parasite culture

The *P*. *falciparum* Cam3.II and V1/S lines were previously engineered to express WT K13 or the C580Y or R539T variants [9]. Parasite lines were maintained in RBCs obtained from Interstate Blood Bank (Memphis, TN) at 3% hematocrit, in RPMI 1640 medium supplemented with gentamicin, hypoxanthine, and Albumax II. Cultures were maintained at 37°C in modular incubator chambers gassed with 5% CO_2_, 5% O_2_ and 90% N_2_. To obtain highly synchronized parasites, predominantly ring-stage cultures were exposed to 5% D-Sorbitol (Sigma-Aldrich) for 15 min at 37°C to remove mature parasites. After 36 hr of subsequent culture, multinucleated schizonts were purified over a 75% Percoll (Sigma-Aldrich) gradient or a magnetic-activated cell sorting (MACS) column (Miltenyi Biotec). Purified schizonts were allowed to invade fresh RBCs for 3 hr, and early rings (0–3 hr post-invasion) were treated with 5% D-Sorbitol to remove any remaining schizonts. These synchronized rings were then used for stage-specific assays. Synchronized trophozoites were harvested 24 hr later.

### *In vitro* determination of IC_50_ levels

IC_50_ values were determined by testing parasites against two-fold serial dilutions of antimalarial compounds [48]. Compounds were tested in duplicate in 96-well plates, with the final volume per well equal to 200 μL. Parasites were seeded at 0.2% parasitemia and 1% hematocrit. Parasites were either continuously exposed to drugs for 72 hr, or pulsed with drug for 3 hr followed by three rounds of washing to remove drug and a further 69 hr of culture in drug-free medium. Washes were performed by centrifuging 96-well plates at 800×g for 2 minutes to pellet cells, removing drug-containing medium, and resuspending in an equal volume of fresh, drug-free medium. On the third wash, cultures were transferred to a new 96-well plate. Removal of media and resuspension were performed on a Freedom Evo 100 liquid-handling instrument (Tecan). After 72 hr, parasites were stained with 1×SYBR Green and 100 nM Mitotracker Deep Red (ThermoFisher) and parasitemias were measured on a BD Accuri C6 Plus Flow Cytometer with a HyperCyt attachment sampling 10,0000–20,000 events per well [78]. Data were analyzed using FlowJo and IC_50_ values were derived using nonlinear regression analysis (GraphPad Prism).

### Stage-specificity studies

Highly-synchronized parasites were exposed to WLL (150 nM), WLW (2000 nM), DHA (150 nM), or DMSO vehicle control for 1 hr followed by drug washout and further culture. All stage-specificity tests were concluded 72 hr from the start of the experiment. Assays were performed in duplicate. Parasites were prepared by synchronizing ring stages with 5% D-Sorbitol and later isolating the schizonts over a 75% Percoll gradient. These schizonts were allowed to reinvade fresh RBCs at 2% hematocrit. Parasites were tested as purified schizonts, early rings, mid rings, late rings or trophozoites (~45–47, 0–3, 10–13, 18–21 and 24–27 hr post-invasion, respectively), and exposed to drug or DMSO vehicle control in 1 mL volumes. Parasites were seeded at 0.5% parasitemia and 1% hematocrit in a 48-well plate.

Drug-treated parasites were washed as previously described for the RSA_0-3h_ [9]. Briefly, 1 mL cultures were transferred to 15 mL conical tubes, centrifuged at 800×g for 5 minutes to pellet cells, and the culture medium containing drug was carefully removed. Cells were subsequently resuspended in 10 mL drug-free medium, centrifuged, and the wash medium removed. Finally, cells were resuspended in 1 mL of fresh drug-free medium, transferred to a new well, and returned to standard culture conditions for the duration of the assay. Parasitemias were measured by flow cytometry and the survival of drug-treated parasites was calculated as a percent of DMSO-treated control cultures.

### Validation of washing protocol for stage-specificity studies

Uninfected RBCs were exposed to WLL (150 nM), WLW (2000 nM), DHA (150 nM) or DMSO vehicle control for 1 hr, followed by drug washout exactly as above. For each treatment, 20 μL of packed RBCs were exposed to drug, equivalent to 1 mL of a 2% hematocrit complete media and blood mixture. Synchronized late trophozoites were purified over a MACS column (Miltenyi Biotec) following initial synchronization with 5% D-Sorbitol, and were exposed to drug-pretreated RBCs at 0.5% parasitemia. Experiments were performed in duplicate. Parasitemias were measured by flow cytometry 48 hr later and percent growth was calculated relative to DMSO-treated control cultures.

### *In vitro* generation of drug-resistant lines

To select for WLL- or WLW-resistant parasites, triplicate flasks of 2×10^9^ parasites, each with a starting parasitemia below 2%, were exposed to these compounds at concentrations of 3× or 5× their IC_50_ values. Selections were performed on Cam3.II K13^WT^, Cam3.II K13^C580Y^, V1/S K13^WT^, and V1/S K13^C580Y^. Drug-containing media was refreshed every day for the first six days, then every 2–4 days. RBCs were replenished every seven days and volumes were reduced by half every week starting on day 14. Cultures were monitored by Giemsa staining and microscopy every day until parasites cleared, then monitored two to three times per week to detect recrudescence. Selections were maintained for 60 days or until recrudescent parasites were observed microscopically.

### Illumina-based whole-genome sequencing

gDNA was prepared from 0.05% saponin-lysed cultures using a DNeasy Blood and Tissue Kit (Qiagen). To prepare the sequencing libraries, gDNA was fragmented and amplified with the Nextera XT kit (Cat. No FC-131–1024, Illumina) using the standard dual index protocol. Libraries were sequenced on an Illumina HiSeq 2500 using the RapidRun mode [34]. Sequence reads (2×100 bp) were aligned to the *P*. *falciparum* 3D7 reference genome (PlasmoDB v. 13.0) using the Platypus pipeline and the Genome Analysis Toolkit’s (GATK) HaplotypeCaller was used to call single nucleotide variants (SNVs), copy number variants (CNVs), or insertion/deletions (INDELs) [79,80]. SNVs were filtered out if they met the following criteria: ReadPosRankSum >8.0 or <−8.0, QUAL <500, Quality by Depth (QD) <2.0, Mapping Quality Rank Sum <−12.5, or filtered depth (DP) <7. INDELS were filtered out if they met the following criteria: ReadPosRankSum <−20, QUAL <500, QD <2, or DP <7. We also removed mutations where the read coverage was <5. Variants were annotated using SnpEff [81]. In instances where drug-selected lines showed sequence heterogeneity, clones were generated by limiting dilution and the candidate SNVs were confirmed by targeted gene sequencing.

### Activity-based probe labeling of the *P*. *falciparum* proteasome

Proteasome activity was profiled using the BMV037 active-site probe [26,38]. Synchronized late schizonts were harvested and lysates were prepared by adding equal volumes of hypotonic lysis buffer (50 mM Tris pH 7.4, 5 mM MgCl_2_, 1 mM DTT) to parasite pellets. Lysates were incubated on ice for 1 hr, with occasional vortexing, then spun at 13,000×rpm for 15 min to recover supernatants. Protein concentrations were determined using a Bradford assay (Pierce). Lysates (10 μg) were pre-incubated with each inhibitor for 1 hr at 37°C prior to adding 10 μM BMV037 for a further 2 hr at 37°C. Samples were denatured by adding SDS sample buffer, then briefly boiled and electrophoresed on a 12% SDS-PAGE gel. Gels were scanned on the Cy5 channel on a Typhoon Scanner (GE Healthcare). IC_50_ values were calculated for each inhibitor for β2 and β5 (WLL) or β2 only (WLW) by quantifying labeled subunits using ImageJ and normalizing to mock-treated DMSO controls. For these labeling studies with parasite lysate, inhibitor concentrations (0.5 to 50 μM) were selected based on an earlier study with BMV037 [21].

### Molecular dynamics and modeling of proteasome mutations

The cryo-EM derived atomic structure of the *P*. *falciparum* 20S proteasome (PDB accession code 5FMG) was used to model the following mutations *in silico*: A20S in β5, A117V in β6, S208L in β6, C31Y in β2, C31F β2, and A49E in β2. These models were used to dock WLL or WLW into the mutant β5 and β2 subunits, respectively. For the β5 and β6 mutations, the structural consequences of these mutations were examined using molecular dynamics simulations on models containing only the active site subunits without the WLL ligand. Standard procedures in the AMBER 14 software package (PME MD protocol) were used, namely generation of topology files for proteins and preparation of AMBER input data using the LeaP module, development of the hydration model in a periodic water box (TIP3P model of water for explicit solvent), thermodynamic equilibration of the system and the solvent, and molecular dynamics calculations at 37°C using the AMBER ff99bsc0 force field with a simulation time of 10 ns [82]. The most frequent states for each simulation were used to infer the effects of mutations on the binding of WLL. In contrast, the β2 mutations were superposed onto the *P*. *falciparum* 20S β2β3 dimer structure (PDB accession code 5FMG). These mutated models were prepared using the Protein Preparation Wizard in Maestro (Schrödinger). Structures were minimized with a harmonic constraint on all heavy atoms (maximum RMSD of 0.3 Å). The binding poses of WLW in the 20S β2 mutants obtained by superposition were then refined by local minimization with Prime (Schrödinger). This local minimization included WLW as well as all protein residues within 5 Å of the ligand, and used the variable-dielectric Generalized Born solvation model. Graphic representations of all resulting models were prepared using the PyMOL Molecular Graphics System (Schrödinger).

### Isobologram analyses

Assays were performed with fixed ratios of drug combinations (1:0, 4:1, 2:1, 1:1, 1:2, 1:4, and 0:1), tested in duplicate [45]. Combinations were prepared from 16×IC_50_ drug stocks and tested across a range of two-fold dilutions. Assays were conducted with asynchronous cultures exposed for 72 hr or with tightly synchronized rings or trophozoites (tested at 0–3 hr or 24–27 hr post-invasion, respectively) exposed for 3 hr. Post-pulse drug washouts were conducted as described above for *in vitro* determination of IC_50_ levels. IC_50_ values were derived for each compound tested alone, and fractional IC_50_ (FIC_50_) values were determined for each compound tested in combination (FIC_50_ = IC_50_ of the drug alone/IC_50_ of the drug in combination) and plotted for each drug combination. For each combination, the mean of the sums of FIC_50_ values at each combination (mean ΣFIC_50_) was also calculated and the results illustrated using heat maps.

### Ethics Statement

Human RBCs used in this study were purchased from the Interstate Blood Bank (Memphis, TN) as blood from anonymized donors. Approval to use this material for *P*. *falciparum in vitro* culture has been granted by the Columbia University Medical Center Institutional Review Board, which has classified this work as not being human subjects research.

## Supporting information

S1 Fig WLL synergizes with multiple classes of structurally and functionally diverse antimalarials. Isobolograms of WLL and (in descending order) DHA, OZ439, MB, b-AP15 and ES_I_ tested on asynchronous parasites and highly synchronized rings and trophozoites. Cam3.II K13^WT^ or Cam3.II K13^C580Y^ parasites were exposed to compounds mixed at fixed ratios of their individual IC_50_ values (1:0, 4:1, 2:1, 1:1, 1;2, 1:4, 0:1). Asynchronous parasites were exposed for 72 hr and parasitemias were determined by flow cytometry. Highly synchronized rings (0–3 hr post-invasion) or trophozoites (tested 24 hr later) were exposed for 3 hr, followed by drug washouts and continued culture for 69 hr in drug-free media. Fractional IC_50_ (FIC_50_) values were plotted for each combination and ratio of drugs tested and results were compared against a hypothetical isobole line illustrating a perfectly additive interaction (dashed line). Data show results of two independent isobologram assays (shown in different shades), each performed in duplicate, tested against Cam3.II K13^WT^ (blue) and Cam3.II K13^C580Y^ (red). Synergy is evidenced by individual FIC_50_ pairwise values falling below the dashed line of additive interactions (FIC_50_ = 1). DHA, dihydroartemisinin; ES_I_, eeyarestatin I; MB, methylene blue.
(PDF)

S2 Fig WLL and WLW antagonize distinct classes of antimalarial compounds. Heat maps of interactions between the WLL or WLW proteasome inhibitors and distinct antimalarial agents. Assays used the Cam3.II K13^WT^ and Cam3.II K13^C580Y^ lines. Parasites were exposed to compounds mixed at fixed ratios of their individual IC_50_ values (1:0, 4:1, 2:1, 1:1, 1;2, 1:4, 0:1). Asynchronous parasites were exposed for 72 hr and parasitemias were determined by flow cytometry. Values represent the mean of the sums of the FIC_50_ values over the five fixed ratios of the two test compounds (excluding the 1:0 and 0:1 points). Assays were conducted on two to four independent occasions in duplicate. CHX, Cyclohexamide; HFG, halofuginone. Means of the sums of FIC_50_ (mean ΣFIC_50_) values are reported in **S13 Table**.
(PDF)

S1 Table Geographic origin and drug resistance genotypes of *Plasmodium falciparum* lines. (PDF)

S2 Table WLL and WLW 72 hr IC_50_ values. (PDF)

S3 Table WLL and WLW 3 hr IC_50_ values. (PDF)

S4 Table Mean percent survival of synchronized parasites exposed for 1 hr to proteasome inhibitors or DHA. (PDF)

S5 Table Mean percent growth of synchronized trophozoites exposed to proteasome inhibitor- or DHA-pretreated RBCs. (PDF)

S6 Table Whole-genome sequence analysis of WLL- and WLW-pressured parasite lines. (PDF)

S7 Table Whole-genome sequence analysis of WLL- and WLW-pressured parasite lines by mutation. (PDF)

S8 Table IC_50_ values of *P. falciparum* lines selected for resistance to WLL or WLW. (PDF)

S9 Table IC_90_ values of *P. falciparum* lines selected for resistance to WLL or WLW. (PDF)

S10 Table IC_50_ values of activity-based probe profiling of WLL- or WLW-resistant lines. (PDF)

S11 Table Compounds used for isobologram analyses. (PDF)

S12 Table Fractional IC_50_ values from isobologram analyses of compounds tested on asynchronous parasites, synchronized rings and synchronized trophozoites, presented as the means of the FIC_50_ sums. (PDF)

S13 Table Fractional IC_50_ values from isobologram analyses on asynchronous parasites only, presented as the means of the FIC_50_ sums. (PDF)

## References

[1] World Health Organization. WHO status report on artemisinin resistance and ACT efficacy. 2018. https://www.who.int/malaria/publications/atoz/artemisinin-resistance-august2018/en

[2] White NJ. Qinghaosu (artemisinin): the price of success. Science. 2008; 320: 330–4. 10.1126/science.1155165 18420924

[3] Tu Y. The discovery of artemisinin (qinghaosu) and gifts from Chinese medicine. Nat Med. 2011; 17: 1217–20. 10.1038/nm.2471 21989013

[4] Ashley EA, Dhorda M, Fairhurst RM, Amaratunga C, Lim P, et al Spread of artemisinin resistance in P*lasmodium falciparum* malaria. N Engl J Med. 2014; 371: 411–23. 10.1056/NEJMoa1314981 25075834 PMC4143591

[5] Imwong M, Suwannasin K, Kunasol C, Sutawong K, Mayxay M, et al The spread of artemisinin-resistant *Plasmodium falciparum* in the Greater Mekong subregion: a molecular epidemiology observational study. Lancet Infect Dis. 2017; 17: 491–7. 10.1016/S1473-3099(17)30048-8 28161569 PMC5406483

[6] Das S, Saha B, Hati AK, Roy S. Evidence of artemisinin-resistant *Plasmodium falciparum* malaria in Eastern India. N Engl J Med. 2018; 379: 1962–4. 10.1056/NEJMc1713777 30428283

[7] Ariey F, Witkowski B, Amaratunga C, Beghain J, Langlois AC, et al A molecular marker of artemisinin-resistant *Plasmodium falciparum* malaria. Nature. 2014; 505: 50–5. 10.1038/nature12876 24352242 PMC5007947

[8] Ghorbal M, Gorman M, Macpherson CR, Martins RM, Scherf A, et al Genome editing in the human malaria parasite *Plasmodium falciparum* using the CRISPR-Cas9 system. Nat Biotechnol. 2014; 32: 819–21. 10.1038/nbt.2925 24880488

[9] Straimer J, Gnadig NF, Witkowski B, Amaratunga C, Duru V, et al K13-propeller mutations confer artemisinin resistance in *Plasmodium falciparum* clinical isolates. Science. 2015; 347: 428–31. 10.1126/science.1260867 25502314 PMC4349400

[10] Petroski MD, Deshaies RJ. Function and regulation of cullin-RING ubiquitin ligases. Nat Rev Mol Cell Biol. 2005; 6: 9–20. 10.1038/nrm1547 15688063

[11] Genschik P, Sumara I, Lechner E. The emerging family of CULLIN3-RING ubiquitin ligases (CRL3s): cellular functions and disease implications. EMBO J. 2013; 32: 2307–20. 10.1038/emboj.2013.173 23912815 PMC3770339

[12] Malaria GEN *Plasmodium falciparum* Community Project. Genomic epidemiology of artemisinin resistant malaria. Elife. 2016; 5: 10.7554/eLife.08714 26943619 PMC4786412

[13] Menard D, Khim N, Beghain J, Adegnika AA, Shafiul-Alam M, et al A worldwide map of *Plasmodium falciparum* K13-propeller polymorphisms. N Engl J Med. 2016; 374: 2453–64. 10.1056/NEJMoa1513137 27332904 PMC4955562

[14] Mok S, Ashley EA, Ferreira PE, Zhu L, Lin Z, et al Population transcriptomics of human malaria parasites reveals the mechanism of artemisinin resistance. Science. 2015; 347: 431–5. 10.1126/science.1260403 25502316 PMC5642863

[15] Dogovski C, Xie SC, Burgio G, Bridgford J, Mok S, et al Targeting the cell stress response of *Plasmodium falciparum* to overcome artemisinin resistance. PLoS Biol. 2015; 13: e1002132 10.1371/journal.pbio.1002132 25901609 PMC4406523

[16] Bridgford JL, Xie SC, Cobbold SA, Pasaje CFA, Herrmann S, et al Artemisinin kills malaria parasites by damaging proteins and inhibiting the proteasome. Nat Commun. 2018; 9: 3801 10.1038/s41467-018-06221-1 30228310 PMC6143634

[17] Voges D, Zwickl P, Baumeister W. The 26S proteasome: a molecular machine designed for controlled proteolysis. Annu Rev Biochem. 1999; 68: 1015–68. 10.1146/annurev.biochem.68.1.1015 10872471

[18] Naujokat C, Hoffmann S. Role and function of the 26S proteasome in proliferation and apoptosis. Lab Invest. 2002; 82: 965–80. 12177235 10.1097/01.lab.0000022226.23741.37

[19] Gantt SM, Myung JM, Briones MR, Li WD, Corey EJ, et al Proteasome inhibitors block development of *Plasmodium spp*. Antimicrob Agents Chemother. 1998; 42: 2731–8. 9756786 10.1128/aac.42.10.2731 PMC105928

[20] Czesny B, Goshu S, Cook JL, Williamson KC. The proteasome inhibitor epoxomicin has potent *Plasmodium falciparum* gametocytocidal activity. Antimicrob Agents Chemother. 2009; 53: 4080–5. 10.1128/AAC.00088-09 19651911 PMC2764187

[21] Li H, O'Donoghue AJ, van der Linden WA, Xie SC, Yoo E, et al Structure- and function-based design of *Plasmodium*-selective proteasome inhibitors. Nature. 2016; 530: 233–6. 10.1038/nature16936 26863983 PMC4755332

[22] LaMonte GM, Almaliti J, Bibo-Verdugo B, Keller L, Zou BY, et al Development of a potent inhibitor of the *Plasmodium* proteasome with reduced mammalian toxicity. J Med Chem. 2017; 60: 6721–32. 10.1021/acs.jmedchem.7b00671 28696697 PMC5554889

[23] Kirkman LA, Zhan W, Visone J, Dziedziech A, Singh PK, et al Antimalarial proteasome inhibitor reveals collateral sensitivity from intersubunit interactions and fitness cost of resistance. Proc Natl Acad Sci USA. 2018; 115: E6863–E70. 10.1073/pnas.1806109115 29967165 PMC6055138

[24] Yoo E, Stokes BH, de Jong H, Vanaerschot M, Kumar T, et al Defining the determinants of specificity of *Plasmodium* proteasome inhibitors. J Am Chem Soc. 2018; 140: 11424–37. 10.1021/jacs.8b06656 30107725 PMC6407133

[25] Xie SC, Gillett DL, Spillman NJ, Tsu C, Luth MR, et al Target validation and identification of novel boronate inhibitors of the *Plasmodium falciparum* proteasome. J Med Chem. 2018; 61: 10053–66. 10.1021/acs.jmedchem.8b01161 30373366 PMC6257627

[26] Li H, van der Linden WA, Verdoes M, Florea BI, McAllister FE, et al Assessing subunit dependency of the *Plasmodium* proteasome using small molecule inhibitors and active site probes. ACS Chem Biol. 2014; 9: 1869–76. 10.1021/cb5001263 24918547 PMC4136710

[27] Klonis N, Crespo-Ortiz MP, Bottova I, Abu-Bakar N, Kenny S, et al Artemisinin activity against *Plasmodium falciparum* requires hemoglobin uptake and digestion. Proc Natl Acad Sci USA. 2011; 108: 11405–10. 10.1073/pnas.1104063108 21709259 PMC3136263

[28] Klonis N, Xie SC, McCaw JM, Crespo-Ortiz MP, Zaloumis SG, et al Altered temporal response of malaria parasites determines differential sensitivity to artemisinin. Proc Natl Acad Sci USA. 2013; 110: 5157–62. 10.1073/pnas.1217452110 23431146 PMC3612604

[29] Xie SC, Dogovski C, Hanssen E, Chiu F, Yang T, et al Haemoglobin degradation underpins the sensitivity of early ring stage *Plasmodium falciparum* to artemisinins. J Cell Sci. 2016; 129: 406–16. 10.1242/jcs.178830 26675237 PMC4732288

[30] Witkowski B, Amaratunga C, Khim N, Sreng S, Chim P, et al Novel phenotypic assays for the detection of artemisinin-resistant *Plasmodium falciparum* malaria in Cambodia: *in-vitro* and *ex-vivo* drug-response studies. Lancet Infect Dis. 2013; 13: 1043–9. 10.1016/S1473-3099(13)70252-4 24035558 PMC5015432

[31] Siriwardana A, Iyengar K, Roepe PD. Endoperoxide drug cross-resistance patterns for *Plasmodium falciparum* exhibiting an artemisinin delayed-clearance phenotype. Antimicrob Agents Chemother. 2016; 60: 6952–6. 10.1128/AAC.00857-16 27600038 PMC5075116

[32] Straimer J, Gnadig NF, Stokes BH, Ehrenberger M, Crane AA, et al *Plasmodium falciparum* K13 mutations differentially impact ozonide susceptibility and parasite fitness *in vitro*. MBio. 2017; 8: e00172–17. 10.1128/mBio.00172-17 28400526 PMC5388803

[33] Lee AH, Fidock DA. Evidence of a mild mutator phenotype in Cambodian *Plasmodium falciparum* malaria parasites. PLoS One. 2016; 11: e0154166 10.1371/journal.pone.0154166 27100094 PMC4839739

[34] Cowell AN, Istvan ES, Lukens AK, Gomez-Lorenzo MG, Vanaerschot M, et al Mapping the malaria parasite druggable genome by using *in vitro* evolution and chemogenomics. Science. 2018; 359: 191–9. 10.1126/science.aan4472 29326268 PMC5925756

[35] Nair S, Miller B, Barends M, Jaidee A, Patel J, et al Adaptive copy number evolution in malaria parasites. PLoS Genet. 2008; 4: e1000243 10.1371/journal.pgen.1000243 18974876 PMC2570623

[36] Kumpornsin K, Modchang C, Heinberg A, Ekland EH, Jirawatcharadech P, et al Origin of robustness in generating drug-resistant malaria parasites. Mol Biol Evol. 2014; 31: 1649–60. 10.1093/molbev/msu140 24739308 PMC4069624

[37] Day KP, Karamalis F, Thompson J, Barnes DA, Peterson C, et al Genes necessary for expression of a virulence determinant and for transmission of *Plasmodium falciparum* are located on a 0.3-megabase region of chromosome 9. Proc Natl Acad Sci USA. 1993; 90: 8292–6. 10.1073/pnas.90.17.8292 8367496 PMC47335

[38] Verdoes M, Florea BI, Menendez-Benito V, Maynard CJ, Witte MD, et al A fluorescent broad-spectrum proteasome inhibitor for labeling proteasomes *in vitro* and *in vivo*. Chem Biol. 2006; 13: 1217–26. 10.1016/j.chembiol.2006.09.013 17114003

[39] Hewings DS, Flygare JA, Wertz IE, Bogyo M. Activity-based probes for the multicatalytic proteasome. FEBS J. 2017; 284: 1540–54. 10.1111/febs.14016 28107776

[40] Li H, Bogyo M, da Fonseca PC. The cryo-EM structure of the *Plasmodium falciparum* 20S proteasome and its use in the fight against malaria. FEBS J. 2016; 283: 4238–43. 10.1111/febs.13780 27286897 PMC5140733

[41] Jourdan J, Matile H, Reift E, Biehlmaier O, Dong Y, et al Monoclonal antibodies that recognize the alkylation signature of antimalarial ozonides OZ277 (Arterolane) and OZ439 (Artefenomel). ACS Infect Dis. 2016; 2: 54–61. 10.1021/acsinfecdis.5b00090 26819968 PMC4718528

[42] Ismail HM, Barton VE, Panchana M, Charoensutthivarakul S, Biagini GA, et al A click chemistry-based proteomic approach reveals that 1,2,4-Trioxolane and artemisinin antimalarials share a common protein alkylation profile. Angew Chem Int Ed Engl. 2016; 55: 6401–5. 10.1002/anie.201512062 27089538 PMC4934138

[43] Haldar K, Bhattacharjee S, Safeukui I. Drug resistance in *Plasmodium*. Nat Rev Microbiol. 2018; 16: 156–70. 10.1038/nrmicro.2017.161 29355852 PMC6371404

[44] Canfield CJ, Pudney M, Gutteridge WE. Interactions of atovaquone with other antimalarial drugs against *Plasmodium falciparum in vitro*. Exp Parasitol. 1995; 80: 373–81. 10.1006/expr.1995.1049 7729473

[45] Moura PA, Dame JB, Fidock DA. Role of *Plasmodium falciparum* digestive vacuole plasmepsins in the specificity and antimalarial mode of action of cysteine and aspartic protease inhibitors. Antimicrob Agents Chemother. 2009; 53: 4968–78. 10.1128/AAC.00882-09 19752273 PMC2786340

[46] Pereira MR, Henrich PP, Sidhu AB, Johnson D, Hardink J, et al *In vivo* and *in vitro* antimalarial properties of azithromycin-chloroquine combinations that include the resistance reversal agent amlodipine. Antimicrob Agents Chemother. 2011; 55: 3115–24. 10.1128/AAC.01566-10 21464242 PMC3122405

[47] Sonoiki E, Ng CL, Lee MC, Guo D, Zhang YK, et al A potent antimalarial benzoxaborole targets a *Plasmodium falciparum* cleavage and polyadenylation specificity factor homologue. Nat Commun. 2017; 8: 14574 10.1038/ncomms14574 28262680 PMC5343452

[48] Ng CL, Siciliano G, Lee MC, de Almeida MJ, Corey VC, et al CRISPR-Cas9-modified *pfmdr1* protects *Plasmodium falciparum* asexual blood stages and gametocytes against a class of piperazine-containing compounds but potentiates artemisinin-based combination therapy partner drugs. Mol Microbiol. 2016; 101: 381–93. 10.1111/mmi.13397 27073104 PMC4958522

[49] Geary TG, Jensen JB. Effects of antibiotics on *Plasmodium falciparum in vitro*. Am J Trop Med Hyg. 1983; 32: 221–5. 6340539 10.4269/ajtmh.1983.32.221

[50] Baragana B, Hallyburton I, Lee MC, Norcross NR, Grimaldi R, et al A novel multiple-stage antimalarial agent that inhibits protein synthesis. Nature. 2015; 522: 315–20. 10.1038/nature14451 26085270 PMC4700930

[51] Coteron JM, Marco M, Esquivias J, Deng X, White KL, et al Structure-guided lead optimization of triazolopyrimidine-ring substituents identifies potent *Plasmodium falciparum* dihydroorotate dehydrogenase inhibitors with clinical candidate potential. J Med Chem. 2011; 54: 5540–61. 10.1021/jm200592f 21696174 PMC3156099

[52] Herman JD, Pepper LR, Cortese JF, Estiu G, Galinsky K, et al The cytoplasmic prolyl-tRNA synthetase of the malaria parasite is a dual-stage target of febrifugine and its analogs. Sci Transl Med. 2015; 7: 288ra77 10.1126/scitranslmed.aaa3575 25995223 PMC4675670

[53] Rottmann M, McNamara C, Yeung BK, Lee MC, Zou B, et al Spiroindolones, a potent compound class for the treatment of malaria. Science. 2010; 329: 1175–80. 10.1126/science.1193225 20813948 PMC3050001

[54] Blasco B, Leroy D, Fidock DA. Antimalarial drug resistance: linking *Plasmodium falciparum* parasite biology to the clinic. Nat Med. 2017; 23: 917–28. 10.1038/nm.4381 28777791 PMC5747363

[55] Phillips MA, Burrows JN, Manyando C, van Huijsduijnen RH, Van Voorhis WC, et al Malaria. Nat Rev Dis Primers. 2017; 3: 17050 10.1038/nrdp.2017.50 28770814

[56] Llanos-Cuentas A, Casapia M, Chuquiyauri R, Hinojosa JC, Kerr N, et al Antimalarial activity of single-dose DSM265, a novel *Plasmodium* dihydroorotate dehydrogenase inhibitor, in patients with uncomplicated *Plasmodium falciparum* or *Plasmodium vivax* malaria infection: a proof-of-concept, open-label, phase 2a study. Lancet Infect Dis. 2018; 18: 874–83. 10.1016/S1473-3099(18)30309-8 29909069 PMC6060173

[57] Wang J, Lin Q. Chemical proteomics approach reveals the direct targets and the heme-dependent activation mechanism of artemisinin in *Plasmodium falciparum* using an artemisinin-based activity probe. Microb Cell. 2016; 3: 230–1. 10.15698/mic2016.05.503 28357359 PMC5349152

[58] Ismail HM, Barton V, Phanchana M, Charoensutthivarakul S, Wong MH, et al Artemisinin activity-based probes identify multiple molecular targets within the asexual stage of the malaria parasites *Plasmodium falciparum* 3D7. Proc Natl Acad Sci USA. 2016; 113: 2080–5. 10.1073/pnas.1600459113 26858419 PMC4776496

[59] Phyo AP, Jittamala P, Nosten FH, Pukrittayakamee S, Imwong M, et al Antimalarial activity of artefenomel (OZ439), a novel synthetic antimalarial endoperoxide, in patients with *Plasmodium falciparum* and *Plasmodium vivax* malaria: an open-label phase 2 trial. Lancet Infect Dis. 2016; 16: 61–9. 10.1016/S1473-3099(15)00320-5 26448141 PMC4700386

[60] Macintyre F, Adoke Y, Tiono AB, Duong TT, Mombo-Ngoma G, et al A randomised, double-blind clinical phase II trial of the efficacy, safety, tolerability and pharmacokinetics of a single dose combination treatment with artefenomel and piperaquine in adults and children with uncomplicated *Plasmodium falciparum* malaria. BMC Med. 2017; 15: 181 10.1186/s12916-017-0940-3 28988541 PMC5632828

[61] Adjalley SH, Johnston GL, Li T, Eastman RT, Ekland EH, et al Quantitative assessment of *Plasmodium falciparum* sexual development reveals potent transmission-blocking activity by methylene blue. Proc Natl Acad Sci USA. 2011; 108: E1214–23. 10.1073/pnas.1112037108 22042867 PMC3223476

[62] Coulibaly B, Pritsch M, Bountogo M, Meissner PE, Nebie E, et al Efficacy and safety of triple combination therapy with artesunate-amodiaquine-methylene blue for *falciparum* malaria in children: a randomized controlled trial in Burkina Faso. J Infect Dis. 2015; 211: 689–97. 10.1093/infdis/jiu540 25267980

[63] Siciliano G, Santha Kumar TR, Bona R, Camarda G, Calabretta MM, et al A high susceptibility to redox imbalance of the transmissible stages of *Plasmodium falciparum* revealed with a luciferase-based mature gametocyte assay. Mol Microbiol. 2017; 104: 306–18. 10.1111/mmi.13626 28118506 PMC5380559

[64] Meierjohann S, Walter RD, Muller S. Regulation of intracellular glutathione levels in erythrocytes infected with chloroquine-sensitive and chloroquine-resistant *Plasmodium falciparum*. Biochem J. 2002; 368: 761–8. 10.1042/BJ20020962 12225291 PMC1223037

[65] Muller S. Role and regulation of glutathione metabolism in *Plasmodium falciparum*. Molecules. 2015; 20: 10511–34. 10.3390/molecules200610511 26060916 PMC6272303

[66] Akoachere M, Buchholz K, Fischer E, Burhenne J, Haefeli WE, et al *In vitro* assessment of methylene blue on chloroquine-sensitive and -resistant *Plasmodium falciparum* strains reveals synergistic action with artemisinins. Antimicrob Agents Chemother. 2005; 49: 4592–7. 10.1128/AAC.49.11.4592-4597.2005 16251300 PMC1280165

[67] Coertzen D, Reader J, van der Watt M, Nondaba SH, Gibhard L, et al Artemisone and artemiside are potent panreactive antimalarial agents that also synergize redox imbalance in *Plasmodium falciparum* transmissible gametocyte stages. Antimicrob Agents Chemother. 2018; 62: 10.1128/AAC.02214-17 29866868 PMC6105806

[68] Wang X, D'Arcy P, Caulfield TR, Paulus A, Chitta K, et al Synthesis and evaluation of derivatives of the proteasome deubiquitinase inhibitor b-AP15. Chem Biol Drug Des. 2015; 86: 1036–48. 10.1111/cbdd.12571 25854145 PMC4846425

[69] Wang L, Delahunty C, Fritz-Wolf K, Rahlfs S, Helena Prieto J, et al Characterization of the 26S proteasome network in *Plasmodium falciparum*. Sci Rep. 2015; 5: 17818 10.1038/srep17818 26639022 PMC4671066

[70] D'Arcy P, Brnjic S, Olofsson MH, Fryknas M, Lindsten K, et al Inhibition of proteasome deubiquitinating activity as a new cancer therapy. Nat Med. 2011; 17: 1636–40. 10.1038/nm.2536 22057347

[71] Fiebiger E, Hirsch C, Vyas JM, Gordon E, Ploegh HL, et al Dissection of the dislocation pathway for type I membrane proteins with a new small molecule inhibitor, eeyarestatin. Mol Biol Cell. 2004; 15: 1635–46. 10.1091/mbc.E03-07-0506 14767067 PMC379262

[72] Ruggiano A, Foresti O, Carvalho P. Quality control: ER-associated degradation: protein quality control and beyond. J Cell Biol. 2014; 204: 869–79. 10.1083/jcb.201312042 24637321 PMC3998802

[73] Wang Q, Shinkre BA, Lee JG, Weniger MA, Liu Y, et al The ERAD inhibitor Eeyarestatin I is a bifunctional compound with a membrane-binding domain and a p97/VCP inhibitory group. PLoS One. 2010; 5: e15479 10.1371/journal.pone.0015479 21124757 PMC2993181

[74] Brem GJ, Mylonas I, Bruning A. Eeyarestatin causes cervical cancer cell sensitization to bortezomib treatment by augmenting ER stress and CHOP expression. Gynecol Oncol. 2013; 128: 383–90. 10.1016/j.ygyno.2012.10.021 23107612

[75] Schubert U, Anton LC, Gibbs J, Norbury CC, Yewdell JW, et al Rapid degradation of a large fraction of newly synthesized proteins by proteasomes. Nature. 2000; 404: 770–4. 10.1038/35008096 10783891

[76] Dhingra SK, Redhi D, Combrinck JM, Yeo T, Okombo J, et al A variant PfCRT isoform can contribute to *Plasmodium falciparum* resistance to the first-line partner drug piperaquine. MBio. 2017; 8: e00303–17. 10.1128/mBio.00303-17 28487425 PMC5424201

[77] Drew ME, Banerjee R, Uffman EW, Gilbertson S, Rosenthal PJ, et al *Plasmodium* food vacuole plasmepsins are activated by falcipains. J Biol Chem. 2008; 283: 12870–6. 10.1074/jbc.M708949200 18308731 PMC2442342

[78] Ekland EH, Schneider J, Fidock DA. Identifying apicoplast-targeting antimalarials using high-throughput compatible approaches. FASEB J. 2011; 25: 3583–93. 10.1096/fj.11-187401 21746861 PMC3177575

[79] Manary MJ, Singhakul SS, Flannery EL, Bopp SE, Corey VC, et al Identification of pathogen genomic variants through an integrated pipeline. BMC Bioinformatics. 2014; 15: 63 10.1186/1471-2105-15-63 24589256 PMC3945619

[80] Van der Auwera GA, Carneiro MO, Hartl C, Poplin R, Del Angel G, et al From FastQ data to high confidence variant calls: the Genome Analysis Toolkit best practices pipeline. Curr Protoc Bioinformatics. 2013; 43: 11.01–33. 10.1002/0471250953.bi1110s43 25431634 PMC4243306

[81] Cingolani P, Platts A, Wang le L, Coon M, Nguyen T, et al A program for annotating and predicting the effects of single nucleotide polymorphisms, SnpEff: SNPs in the genome of *Drosophila melanogaster* strain w1118; iso-2; iso-3. Fly. 2012; 6: 80–92. 10.4161/fly.19695 22728672 PMC3679285

[82] Case D, Babin V, Berryman J, Betz R, Cai Q, et al (2014) Amber 2014.

