# Supplementary figures and images for: Covalent Plasmodium falciparum-selective proteasome inhibitors exhibit a low propensity for generating resistance in vitro and synergize with multiple antimalarial agents
Source: PLoS Pathog. 2019 Jun 6;15(6):e1007722. doi: 10.1371/journal.ppat.1007722 (PMC6553790; doi:10.1371/journal.ppat.1007722)

# S1 Figure

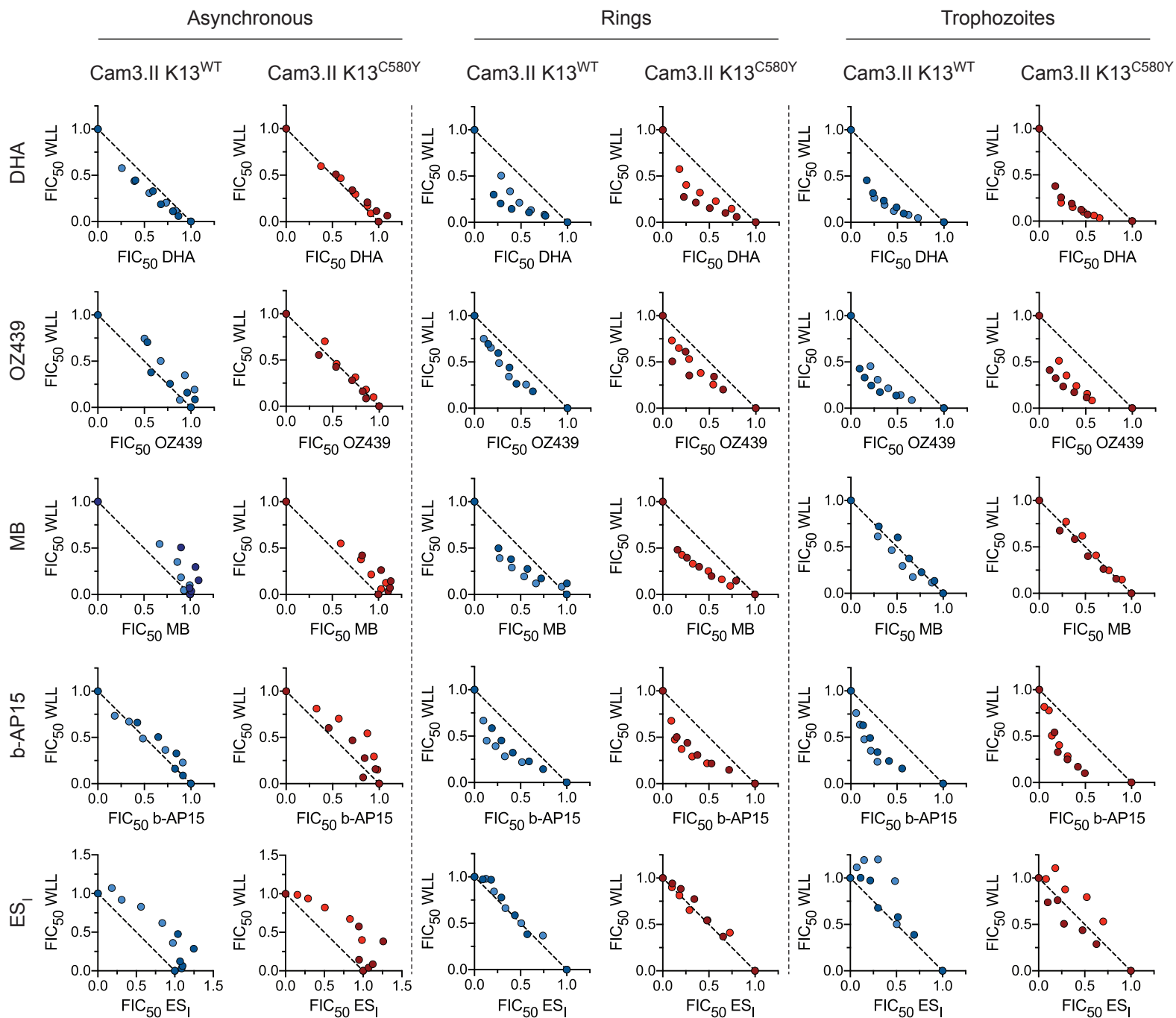

Supplement: S1 Fig — Isobolograms of WLL and (in descending order) DHA, OZ439, MB, b-AP15 and ESI tested on asynchronous parasites and highly synchronized rings and trophozoites. Cam3.II K13WT or Cam3.II K13C580Y parasites were exposed to compounds mixed at fixed ratios of their individual IC50 values (1:0, 4:1, 2:1, 1:1, 1;2, 1:4, 0:1). Asynchronous parasites were exposed for 72 hr and parasitemias were determined by flow cytometry. Highly synchronized rings (0–3 hr post-invasion) or trophozoites (tested 24 hr later) were exposed for 3 hr, followed by drug washouts and continued culture for 69 hr in drug-free media. Fractional IC50 (FIC50) values were plotted for each combination and ratio of drugs tested and results were compared against a hypothetical isobole line illustrating a perfectly additive interaction (dashed line). Data show results of two independent isobologram assays (shown in different shades), each performed in duplicate, tested against Cam3.II K13WT (blue) and Cam3.II K13C580Y (red). Synergy is evidenced by individual FIC50 pairwise values falling below the dashed line of additive interactions (FIC50 = 1). DHA, dihydroartemisinin; ESI, eeyarestatin I; MB, methylene blue. (PDF) [file ppat.1007722.s001.pdf]

# S2 Figure

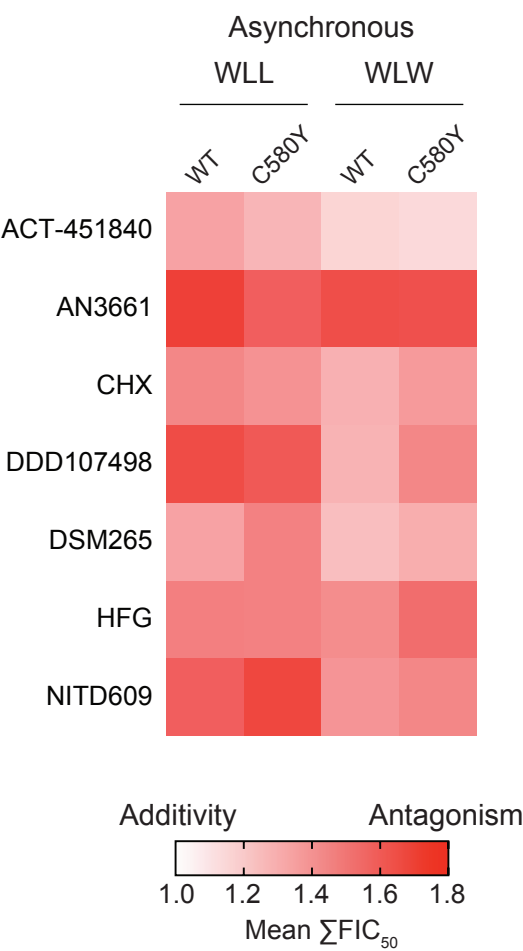

Supplement: S2 Fig — Heat maps of interactions between the WLL or WLW proteasome inhibitors and distinct antimalarial agents. Assays used the Cam3.II K13WT and Cam3.II K13C580Y lines. Parasites were exposed to compounds mixed at fixed ratios of their individual IC50 values (1:0, 4:1, 2:1, 1:1, 1;2, 1:4, 0:1). Asynchronous parasites were exposed for 72 hr and parasitemias were determined by flow cytometry. Values represent the mean of the sums of the FIC50 values over the five fixed ratios of the two test compounds (excluding the 1:0 and 0:1 points). Assays were conducted on two to four independent occasions in duplicate. CHX, Cyclohexamide; HFG, halofuginone. Means of the sums of FIC50 (mean ΣFIC50) values are reported in S13 Table. (PDF) [file ppat.1007722.s002.pdf]
