# Supplementary material for: Covalent Plasmodium falciparum-selective proteasome inhibitors exhibit a low propensity for generating resistance in vitro and synergize with multiple antimalarial agents
Source: PLoS Pathog. 2019 Jun 6;15(6):e1007722. doi: 10.1371/journal.ppat.1007722 (PMC6553790; doi:10.1371/journal.ppat.1007722)
Supplement: S1 Table — (PDF) [file ppat.1007722.s003.pdf]

S1 Table. Geographic origin and drug resistance genotypes of *Plasmodium falciparum* lines.

Parasite[a]	Edited[b]	Original ID	Source	Parental line (year)	*K13*	*pfcrt*[c]	*pfmdr1*	CN[d]	*dhfr*[e]
Cam3.II K13^{WT}	Yes[f]		Fidock lab	W. Cambodia (2010)	WT				
Cam3.II K13^{R539T}	No[g]	RF 967	R. Fairhurst		R539T	Dd2	Y184F	1	Triple
Cam3.II K13^{C580Y}	Yes		Fidock lab		C580Y				
V1/S K13^{WT}	Yes[h]		Fidock lab	Vietnam (1976)	WT				
V1/S K13^{R539T}	Yes	V1/S	Fidock lab		R539T	Dd2	N86Y	1	Quad
V1/S K13^{C580Y}	Yes		Fidock lab		C580Y				

[a]Parasite lines were previously reported by Straimer *et al.* (2015, *Science*).

[b]Zinc-finger nucleases were used to generate gene-edited parasites expressing the wild-type (WT) or mutant alleles along with binding-site mutations.

[c]Dd2: M74I/N75E/K76T/A220S/Q271E/N326S/I356T/R371I.

[d]CN, *pfmdr1* copy number.

[e]Triple, N51I/C59R/S108N; Quad, quadruple N51I/C59R/S108N/I164L.

[f]Cam3.II K13^{R539T} parental line reverted to the WT allele. Previously denoted Cam3.II^{rev} in Straimer *et al.*

[g]Unedited parental line bearing the K13 R539T allele.

[h]Editing control. V1/S parasite bearing the K13 WT allele plus zinc-finger binding-site mutations.
