# Supplementary material for: Covalent Plasmodium falciparum-selective proteasome inhibitors exhibit a low propensity for generating resistance in vitro and synergize with multiple antimalarial agents
Source: PLoS Pathog. 2019 Jun 6;15(6):e1007722. doi: 10.1371/journal.ppat.1007722 (PMC6553790; doi:10.1371/journal.ppat.1007722)
Supplement: S2 Table — (PDF) [file ppat.1007722.s004.pdf]

S2 Table. WLL and WLW 72 hr IC_{50} values.

Parasite line	Inhibitor[a]	Mean ± SEM IC_{50} (nM)[b]	N[c]	*P* value[d]
Cam3.II $K13^{WT}$	WLL	12.1 ± 1.5	9	NA
Cam3.II $K13^{R539T}$	WLL	12.7 ± 1.7	3	0.37 (ns)
Cam3.II $K13^{C580Y}$	WLL	12.9 ± 1.8	8	0.52 (ns)
V1/S $K13^{WT}$	WLL	11.1 ± 2.3	5	NA
V1/S $K13^{R539T}$	WLL	10.9 ± 1.9	3	0.79 (ns)
V1/S $K13^{C580Y}$	WLL	11.8 ± 2.7	5	0.84 (ns)
Cam3.II $K13^{WT}$	WLW	53.4 ± 8.5	7	NA
Cam3.II $K13^{R539T}$	WLW	46.7 ± 8.0	3	0.67 (ns)
Cam3.II $K13^{C580Y}$	WLW	58.6 ± 10.9	6	0.84 (ns)
V1/S $K13^{WT}$	WLW	33.0 ± 4.3	6	NA
V1/S $K13^{R539T}$	WLW	34.0 ± 5.2	3	0.71 (ns)
V1/S $K13^{C580Y}$	WLW	28.7 ± 5.0	6	0.82 (ns)

[a]WLL and WLW are both vinyl sulfones (-vs).

[b]IC_{50} values represent concentrations at which growth was inhibited by 50% in 72 hr assays with asynchronous cultures.

[c]N, number of independent experiments (each with technical duplicates).

[d]Statistics were performed using Mann-Whitney *U* tests, comparing K13-mutant lines to the wild-type (WT) line in the same genetic background.

NA, not applicable (reference line); ns, not significant.
