# Supplementary material for: Covalent Plasmodium falciparum-selective proteasome inhibitors exhibit a low propensity for generating resistance in vitro and synergize with multiple antimalarial agents
Source: PLoS Pathog. 2019 Jun 6;15(6):e1007722. doi: 10.1371/journal.ppat.1007722 (PMC6553790; doi:10.1371/journal.ppat.1007722)
Supplement: S3 Table — (PDF) [file ppat.1007722.s005.pdf]

S3 Table. WLL and WLW 3 hr IC_{50} values.

Parasite line	Inhibitor[a]	Stage[b]	SEM IC_{50} (nM)	N[c]	*P* value[d]
Cam3.II $K13^{WT}$	WLL	Rings	52 ± 9	4	NA
Cam3.II $K13^{C580Y}$	WLL	Rings	54 ± 7	4	0.89 (ns)
Cam3.II $K13^{WT}$	WLL	Trophozoites	422 ± 41	3	NA
Cam3.II $K13^{C580Y}$	WLL	Trophozoites	453 ± 8	3	0.70 (ns)
Cam3.II $K13^{WT}$	WLW	Rings	531 ± 106	4	NA
Cam3.II $K13^{C580Y}$	WLW	Rings	516 ± 83	4	0.70 (ns)
Cam3.II $K13^{WT}$	WLW	Trophozoites	1698 ± 218	3	NA
Cam3.II $K13^{C580Y}$	WLW	Trophozoites	1628 ± 149	3	>0.99 (ns)

[a]WLL and WLW are both vinyl sulfones (-vs).

[b]Cultures were tested in 72 hr assays with 3 hr drug pulses of early rings (0–3 hr post-invasion) or trophozoites (tested 24 hr after the early ring stages), followed by washes.

[c]N, number of independent experiments (each with technical duplicates).

[d]Statistics were performed using Mann-Whitney *U* tests, comparing K13-mutant lines to the wild-type (WT) line in the same genetic background.

NA, not applicable (reference line); ns, not significant.
