# Supplementary material for: Covalent Plasmodium falciparum-selective proteasome inhibitors exhibit a low propensity for generating resistance in vitro and synergize with multiple antimalarial agents
Source: PLoS Pathog. 2019 Jun 6;15(6):e1007722. doi: 10.1371/journal.ppat.1007722 (PMC6553790; doi:10.1371/journal.ppat.1007722)
Supplement: S4 Table — (PDF) [file ppat.1007722.s006.pdf]

S4 Table. Mean percent survival of synchronized parasites exposed for 1 hr to proteasome inhibitors or DHA.

	WLL (150 nM)				WLW (2000 nM)				DHA (150 nM)			
	Cam 3.II K13^{WT}		Cam 3.II K13^{C580Y}		Cam 3.II K13^{WT}		Cam 3.II K13^{C580Y}		Cam 3.II K13^{WT}		Cam 3.II K13^{C580Y}	
Stage[a]	Mean (%)[b]	N[c]	Mean (%)	N	Mean (%)	N	Mean (%)	N	Mean (%)	N	Mean (%)	N
Schizonts (45–48 hpi)	22.6 ± 2.6	3	23.5 ± 4.3	3	6.6 ± 1.0	3	10.7 ± 1.2	3	59.0 ± 5.3	2	71.9 ± 13.6	2
Early rings (0–3 hpi)	49.4 ± 10.9	3	46.2 ± 10.7	3	19.5 ± 4.2	3	18.7 ± 2.9	3	46.8 ± 10.6	2	57.9 ± 5.6	2
Mid rings (10–13 hpi)	93.8 ± 1.0	3	93.2 ± 3.0	3	55.6 ± 9.1	3	53.6 ± 4.3	3	35.4 ± 2.7	2	29.1 ± 3.4	2
Late rings (18–21 hpi)	91.4 ± 1.5	3	80.4 ± 3.6	3	29.6 ± 5.1	3	33.7 ± 3.4	3	18.9 ± 5.6	2	13.5 ± 3.1	2
Trophs (24–27 hpi)	94.0 ± 5.7	3	99.3 ± 13.0	3	23.4 ± 6.3	3	30.1 ± 10.3	3	2.3 ± 0.1	2	4.1 ± 0.6	2

[a]Tightly synchronized parasites were drug-treated for 1 hr, followed by drug washout and incubation in drug-free medium for a further three days.

[b]Percent survival (mean ± SEM) was calculated relative to vehicle-treated control wells.

[c]N, number of independent experiments with technical duplicates.

hpi, hours post-invasion.
