# Supplementary material for: Covalent Plasmodium falciparum-selective proteasome inhibitors exhibit a low propensity for generating resistance in vitro and synergize with multiple antimalarial agents
Source: PLoS Pathog. 2019 Jun 6;15(6):e1007722. doi: 10.1371/journal.ppat.1007722 (PMC6553790; doi:10.1371/journal.ppat.1007722)
Supplement: S5 Table — (PDF) [file ppat.1007722.s007.pdf]

**S5 Table. Mean percent growth of synchronized trophozoites exposed to proteasome inhibitor- or DHA-pretreated RBCs.**

| Parasite line | Treatment[a] | Mean (%) ± SEM[b] | N[c] |
|---|---|---|---|
| Cam 3.II $K13^{WT}$ | 150 nM WLL | 95.5 ± 3.6 | 3 |
| Cam 3.II $K13^{C580Y}$ | 150 nM WLL | 96.5 ± 3.3 | 3 |
| Cam 3.II $K13^{WT}$ | 2000 nM WLW | 78.7 ± 6.8 | 3 |
| Cam 3.II $K13^{C580Y}$ | 2000 nM WLW | 80.7 ± 3.8 | 3 |
| Cam 3.II $K13^{WT}$ | 150 nM DHA | 103.1 ± 1.7 | 3 |
| Cam 3.II $K13^{C580Y}$ | 150 nM DHA | 96.4 ± 2.0 | 3 |

[a]Uninfected RBCs were drug-treated for 1 hr, followed by drug washout. RBCs were then added to magnet-purified late trophozoites for reinvasion. Cultures were continued for an additional 48 hr prior to assesment of parasitemias.

[b]Percent growth (mean ± SEM) was calculated relative to vehicle-treated control wells.

[c]N, number of independent experiments with technical duplicates.
