# Supplementary material for: Covalent Plasmodium falciparum-selective proteasome inhibitors exhibit a low propensity for generating resistance in vitro and synergize with multiple antimalarial agents
Source: PLoS Pathog. 2019 Jun 6;15(6):e1007722. doi: 10.1371/journal.ppat.1007722 (PMC6553790; doi:10.1371/journal.ppat.1007722)
Supplement: S6 Table — (PDF) [file ppat.1007722.s008.pdf]

S6 Table. Whole-genome sequence analysis of WLL- and WLW-pressured parasite lines (page 1 of 4).

				Sample Name	Cam3.II K13[WT]	Cam3.II K13[WT] 3×WLW R1	Cam3.II K13[WT] 3×WLW R2	Cam3.II K13[WT] 3×WLW R3
				Tested Herein?	Yes, reference	Yes (clone A11)	Yes (clone D5)	No
				Shortened Name	NA	RPT4 E380*	RPN6 E266K	NA
				Fold IC_{50} increase[c]	NA	2.2	2.4	ND
				Mean Whole Genome Coverage (×)	81.5	52.2	33.6	63.2
Gene ID	Position	Protein name	Mutation pre-processing[a]	Mutation in mature protein[b]	Reads supporting mutation calls			
PF3D7_1011400	441718	20S β5 subunit	A80S	A20S	**WT**	WT	WT	WT
PF3D7_0518300	760204	20S β6 subunit	A117V	A117V	**WT**	WT	WT	WT
PF3D7_0518300	759931	20S β6 subunit	S208L	S208L	**WT**	WT	WT	WT
PF3D7_1328100	1184422	20S β2 subunit	C72F	C31F	**WT**	WT	WT	WT
PF3D7_1328100	1184422	20S β2 subunit	C72Y	C31Y	**WT**	WT	WT	WT
PF3D7_1328100	1184476	20S β2 subunit	A90E	A49E	**WT**	WT	WT	WT
PF3D7_1306400	297222	19S RPT4	NA	E380*	**WT**	**22/44**	WT	**21/58**
PF3D7_1130400	1169763	19S RPT5	NA	G319S	**WT**	WT	WT	WT
PF3D7_1130400	1169833	19S RPT5	NA	R295S	**WT**	WT	WT	WT
PF3D7_1402300	87088	19S RPN6	NA	E266K	**WT**	**22/58**	**11/27**	**13/64**
PF3D7_0808300	N/A	Ubiquitin regulatory protein	NA	CNV	**No**	No	No	No

[a]Amino acid change in protein prior to proteolytic processing.

[b]Amino acid change in mature protein (post-processing).

[c]These data are reproduced from S8 Table, in which IC_{50} values for WLL and WLW for the nine lines profiled in this study are

CNV, copy number variation; NA, not applicable; ND, not done; WT, wild-type.

S6 Table. Whole-genome sequence analysis of WLL- and WLW-pressured parasite lines (page 2 of 4).

Sample Name	Cam3.II K13^{C580Y}	Cam3.II K13^{C580Y} 3×WLL R1	Cam3.II K13^{C580Y} 3×WLL R2	Cam3.II K13^{C580Y} 5×WLW R1	Cam3.II K13^{C580Y} 3×WLW R1	Cam3.II K13^{C580Y} 3×WLW R2	Cam3.II K13^{C580Y} 3×WLW R3
Tested Herein?	Yes, reference	Yes (clonal)	No	No	Yes (clonal)	Yes (clone C8)	No
Shortened Name	NA	β5 A20S	NA	NA	β2 C31Y	RPT5 G319S	NA
Fold IC_{50} increase[c]	NA	2.4	ND	ND	3.4	1.9	ND
Mean Whole Genome Coverage (×)	42.7	81.2	34.4	16.0	41.2	58.8	58.9
Mutation in mature protein[b]	Reads supporting mutation calls						
A20S	**WT**	**93/97**	**48/48**	WT	WT	WT	WT
A117V	**WT**	WT	WT	WT	WT	WT	WT
S208L	**WT**	WT	WT	WT	WT	WT	WT
C31F	**WT**	WT	WT	WT	WT	WT	**81/85**
C31Y	**WT**	WT	WT	**32/34**	**43/45**	WT	WT
A49E	**WT**	WT	WT	WT	WT	WT	WT
E380*	**WT**	WT	WT	WT	WT	WT	WT
G319S	**WT**	WT	WT	WT	WT	**43/152**	WT
R295S	**WT**	WT	WT	WT	WT	**49/219**	WT
E266K	**WT**	WT	WT	WT	WT	WT	WT
CNV	**No**	No	No	No	No	No	No

S6 Table. Whole-genome sequence analysis of WLL- and WLW-pressured parasite lines (page 3 of 4).

Sample Name	V1/S $K13^{WT}$	V1/S $K13^{WT}$ 3×WLL R3	V1/S $K13^{WT}$ 5×WLW R1	V1S $K13^{WT}$ 5×WLW R2	V1/S $K13^{WT}$ 3×WLW R1	V1/S $K13^{WT}$ 3×WLW R2	V1/S $K13^{WT}$ 3×WLW R3
Tested Herein?	Yes, reference	Yes (clonal)	No	No	No	Yes (clonal)	No
Shortened Name	NA	β6 A117V	NA	NA	NA	β2 C31F	NA
Fold IC_{50} increase[c]	NA	2.3	ND	ND	ND	5.2	ND
Mean Whole Genome Coverage (×)	44.4	54.4	74.0	80.5	47.1	51.5	31.9
Mutation in mature protein[b]	Reads supporting mutation calls						
A20S	**WT**	WT	WT	WT	WT	WT	WT
A117V	**WT**	**77/77**	WT	WT	WT	WT	WT
S208L	**WT**	WT	WT	WT	WT	WT	WT
C31F	**WT**	WT	WT	WT	**16/71**	**73/80**	WT
C31Y	**WT**	WT	WT	**127/128**	**6/71**	WT	**47/72**
A49E	**WT**	WT	**232/234**	WT	WT	WT	**17/72**
E380*	**WT**	WT	WT	WT	WT	WT	WT
G319S	**WT**	WT	WT	WT	WT	WT	WT
R295S	**WT**	WT	WT	WT	WT	WT	WT
E266K	**WT**	WT	WT	WT	WT	WT	WT
CNV	**No**	No	No	No	No	No	No

S6 Table. Whole-genome sequence analysis of WLL- and WLW-pressured parasite lines (page 4 of 4).

Sample Name	V1/S K13^{C580Y}	V1/S K13^{C580Y} 5×WLL R1	V1/S K13^{C580Y} 5×WLL R2	V1/S K13^{C580Y} 3×WLL R1	V1/S K13^{C580Y} 3×WLL R2	V1/S K13^{C580Y} 5×WLW R1	V1/S K13^{C580Y} 5×WLW R2	V1/S K13^{C580Y} 5×WLW R3	V1/S K13^{C580Y} 3×WLL R3	V1/S K13^{C580Y} 3×WLW R1	V1/S K13^{C580Y} 3×WLW R2	V1/S K13^{C580Y} 3×WLW R3
Tested Herein?	Yes, reference	No	No	Yes (clonal)	No	No	No	No	No	No	Yes (clonal)	No
Shortened Name	NA	NA	NA	β6 S208L	NA	NA	NA	NA	NA	NA	β2 A49E	NA
Fold IC_{50} increase[c]	NA	ND	ND	1.2	ND	ND	ND	ND	ND	ND	4.3	ND
Mean Whole Genome Coverage (×)	43.9	76.4	76.4	67.4	40.1	31.4	84.8	20.9	57.0	51.9	57.1	64.6
Mutation in mature protein[b]	Reads supporting mutation calls											
A20S	**WT**	WT	WT	WT	**26/42**	WT	WT	WT	**65/73**	WT	WT	WT
A117V	**WT**	**95/96**	**92/102**	WT	WT	WT	WT	WT	WT	WT	WT	WT
S208L	**WT**	WT	WT	**91/100**	WT	WT	WT	WT	WT	WT	WT	WT
C31F	**WT**	WT	WT	WT	WT	**55/56**	**141/142**	WT	WT	**50/73**	WT	**81/85**
C31Y	**WT**	WT	WT	WT	WT	WT	WT	**35/36**	WT	WT	WT	WT
A49E	**WT**	WT	WT	WT	WT	WT	WT	WT	WT	WT	**194/204**	WT
E380*	**WT**	WT	WT	WT	WT	WT	WT	WT	WT	WT	WT	WT
G319S	**WT**	WT	WT	WT	WT	WT	WT	WT	WT	WT	WT	WT
R295S	**WT**	WT	WT	WT	WT	WT	WT	WT	WT	WT	WT	WT
E266K	**WT**	WT	WT	WT	WT	WT	WT	WT	WT	WT	WT	WT
CNV	**No**	No	No	No	**Present**	No	No	No	No	No	**Present**	No
