# Supplementary material for: Covalent Plasmodium falciparum-selective proteasome inhibitors exhibit a low propensity for generating resistance in vitro and synergize with multiple antimalarial agents
Source: PLoS Pathog. 2019 Jun 6;15(6):e1007722. doi: 10.1371/journal.ppat.1007722 (PMC6553790; doi:10.1371/journal.ppat.1007722)
Supplement: S7 Table — (PDF) [file ppat.1007722.s009.pdf]

S7 Table. Whole-genome sequence analysis of WLL- and WLW-pressured parasite lines by mutation.

Protein name	Gene ID	Mutation in mature protein	Sample Name	Mutation status[a]	Cloned	Tested herein?	Shortened name
20S β5 subunit	PF3D7_1011400	A20S	Cam3.II $K13^{C580Y}$ 3×WLL R1	Clonal	No	Yes	β5 A20S
			Cam3.II $K13^{C580Y}$ 3×WLL R2	Clonal	No	--	--
			V1/S $K13^{C580Y}$ 3×WLL R2	Mixed	No	--	--
			V1/S $K13^{C580Y}$ 3×WLL R3	Mixed	No	--	--
20S β6 subunit	PF3D7_0518300	A117V	V1/S $K13^{C580Y}$ 5×WLL R1	Clonal	No	--	--
			V1/S $K13^{C580Y}$ 5×WLL R2	Clonal	No	--	--
			V1/S $K13^{WT}$ 3×WLL R3	Clonal	No	Yes	β6 A117V
20S β6 subunit	PF3D7_0518300	S208L	V1/S $K13^{C580Y}$ 3×WLL R1	Clonal	No	Yes	β6 208L
20S β2 subunit	PF3D7_1328100	C31F	Cam3.II $K13^{C580Y}$ 3×WLW R3	Clonal	No	--	--
			V1/S $K13^{WT}$ 3×WLW R1[b]	Mixed	No	--	--
			V1/S $K13^{WT}$ 3×WLW R2	Clonal	No	Yes	β2 C31F
			V1/S $K13^{WT}$ 3×WLW R3[c]	Mixed	No	--	--
			V1/S $K13^{C580Y}$ 5×WLW R1	Clonal	No	--	--
			V1/S $K13^{C580Y}$ 3×WLW R1	Mixed	No	--	--
			V1/S $K13^{C580Y}$ 3×WLW R3	Clonal	No	--	--
20S β2 subunit	PF3D7_1328100	C31Y	Cam3.II $K13^{C580Y}$ 5×WLW R1	Clonal	No	--	--
			Cam3.II $K13^{C580Y}$ 3×WLW R1	Clonal	No	Yes	β2 C31Y
			V1/S $K13^{WT}$ 5×WLW R2	Clonal	No	--	--
			V1/S $K13^{WT}$ 3×WLW R1[b]	Mixed	No	--	--
			V1/S $K13^{WT}$ 3×WLW R3[c]	Mixed	No	--	--
			V1/S $K13^{C580Y}$ 5×WLW R2	Clonal	No	--	--
			V1/S $K13^{C580Y}$ 5×WLW R3	Clonal	No	--	--
20S β2 subunit	PF3D7_1328100	A49E	V1/S $K13^{WT}$ 5×WLW R1	Clonal	No	--	--
			V1/S $K13^{C580Y}$ 3×WLW R2	Clonal	No	Yes	β2 A49E
19S RPT4	PF3D7_1306400	E380* (stop)	Cam3.II $K13^{WT}$ 3×WLW R1[d]	Mixed	Yes	Yes (clone A11)[g]	RPT4 E380*
			Cam3.II $K13^{WT}$ 3×WLW R3[e]	Mixed	Yes	--	--
19S RPT5	PF3D7_1130400	R295S	Cam3.II $K13^{C580Y}$ 3×WLW R2[f]	Mixed	Yes	--	--
19S RPT5	PF3D7_1130400	G319S	Cam3.II $K13^{C580Y}$ 3×WLW R2[f]	Mixed	Yes	Yes (clone C8)[g]	RPT5 G319S
19S RPN6	PF3D7_1402300	E266K	Cam3.II $K13^{WT}$ 3×WLW R1[d]	Mixed	Yes	--	--
			Cam3.II $K13^{WT}$ 3×WLW R2	Mixed	Yes	Yes (clone D5)[g]	RPN6 E266K
			Cam3.II $K13^{WT}$ 3×WLW R3[e]	Mixed	Yes	--	--

[a]Clonal indicates that the single mutation is present in ≥90% of reads. Mixed indicates the presence of multiple alleles with frequencies between 20% and 90%. Read counts for reference and alternate alleles are available in S6 Table.

[b,c]Both lines contained a mixture of mutant parasites expressing C31F or C31Y in the 20S β2 subunit.

[d,e]Both lines contained a mixture of mutant parasites expressing the 19S RPT4 E380* stop-gained mutation or the RPN6 E266K mutation.

[f]This line contained a mixture of mutant parasites expressing either the R295S or the G319S mutation in 19S RPT5.

[g]These clones were generated by limiting dilution from the selected lines and are clonal for the designated mutant codon.
