# Supplementary material for: Covalent Plasmodium falciparum-selective proteasome inhibitors exhibit a low propensity for generating resistance in vitro and synergize with multiple antimalarial agents
Source: PLoS Pathog. 2019 Jun 6;15(6):e1007722. doi: 10.1371/journal.ppat.1007722 (PMC6553790; doi:10.1371/journal.ppat.1007722)
Supplement: S8 Table — (PDF) [file ppat.1007722.s010.pdf]

S8 Table. IC_{50} values of *P. falciparum* lines selected for resistance to WLL or WLW.

Parasite line	Parental line	Selection agent	Inhibitor tested	Mean ± SEM IC_{50} (nM)[a]	N[b]	Fold change[c]	*P* value[d]
Cam3.II $K13^{WT}$	--	--	WLL	17.0 ± 1.7	7	NA	NA
RPT4 E380*	Cam3.II $K13^{WT}$	WLW	WLL	15.8 ± 1.7	7	0.9	0.62 (ns)
RPN6 E266K	Cam3.II $K13^{WT}$	WLW	WLL	16.9 ± 2.4	7	1.0	>0.99 (ns)
Cam3.II $K13^{C580Y}$	--	--	WLL	20.8 ± 2.6	6	NA	NA
β5 A20S	Cam3.II $K13^{C580Y}$	WLL	WLL	46.9 ± 3.1	7	2.4	0.0012 (∗∗)
β2 C31Y	Cam3.II $K13^{C580Y}$	WLW	WLL	8.3 ± 0.9	7	0.4	0.0012 (∗∗)
RPT5 G319S	Cam3.II $K13^{C580Y}$	WLW	WLL	19.8 ± 3.6	7	1.0	0.63 (ns)
V1/S $K13^{WT}$	--	--	WLL	14.4 ± 1.1	6	NA	NA
β6 A117V	V1/S $K13^{WT}$	WLL	WLL	33.4 ± 3.7	7	2.3	0.0012 (∗∗)
β2 C31F	V1/S $K13^{WT}$	WLW	WLL	5.5 ± 0.7	6	0.4	0.0022 (∗∗)
V1/S $K13^{C580Y}$	--	--	WLL	14.3 ± 1.7	6	NA	NA
β6 S208L	V1/S $K13^{C580Y}$	WLL	WLL	16.9 ± 1.8	7	1.2	0.45 (ns)
β2 A49E	V1/S $K13^{C580Y}$	WLW	WLL	13.6 ± 1.5	7	1.0	0.84 (ns)
Cam3.II $K13^{WT}$	--	--	WLW	69.5 ± 10.5	6	NA	NA
RPT4 E380*	Cam3.II $K13^{WT}$	WLW	WLW	151.4 ± 20.4	6	2.2	0.0087 (∗∗)
RPN6 E266K	Cam3.II $K13^{WT}$	WLW	WLW	168.2 ± 16.7	6	2.4	0.0043 (∗∗)
Cam3.II $K13^{C580Y}$	--	--	WLW	100.3 ± 5.7	6	NA	NA
β5 A20S	Cam3.II $K13^{C580Y}$	WLL	WLW	32.3 ± 2.5	7	0.3	0.0012 (∗∗)
β2 C31Y	Cam3.II $K13^{C580Y}$	WLW	WLW	340.0 ± 32.0	7	3.4	0.0012 (∗∗)
RPT5 G319S	Cam3.II $K13^{C580Y}$	WLW	WLW	186.4 ± 20.6	5	1.9	0.0087 (∗∗)
V1/S $K13^{WT}$	--	--	WLW	42.7 ± 6.4	6	NA	NA
β6 A117V	V1/S $K13^{WT}$	WLL	WLW	106.4 ± 8.0	7	2.5	0.0012 (∗∗)
β2 C31F	V1/S $K13^{WT}$	WLW	WLW	220.1 ± 16.0	6	5.2	0.0022 (∗∗)
V1/S $K13^{C580Y}$	--	--	WLW	36.7 ± 4.2	6	NA	NA
β6 S208L	V1/S $K13^{C580Y}$	WLL	WLW	41.2 ± 3.7	7	1.1	0.53 (ns)
β2 A49E	V1/S $K13^{C580Y}$	WLW	WLW	156.1 ± 11.9	7	4.3	0.0012 (∗∗)

[a]IC_{50} values represent concentrations at which growth was inhibited by 50% in 72 hr assays with asynchronous cultures.

[b]N, number of independent experiments (each with technical duplicates).

[c]Fold change values represent the ratio of the IC_{50} of the selected line to the IC_{50} of the parental line.

[d]Statistics were performed using Mann-Whitney *U* tests, comparing K13-mutant lines to the wild-type (WT) line in the same genetic background.

NA, not applicable (reference line); ns, not significant; ∗∗ $P<0.01$.
