# Supplementary material for: Covalent Plasmodium falciparum-selective proteasome inhibitors exhibit a low propensity for generating resistance in vitro and synergize with multiple antimalarial agents
Source: PLoS Pathog. 2019 Jun 6;15(6):e1007722. doi: 10.1371/journal.ppat.1007722 (PMC6553790; doi:10.1371/journal.ppat.1007722)
Supplement: S9 Table — (PDF) [file ppat.1007722.s011.pdf]

S9 Table. IC_{90} values of *P. falciparum* lines selected for resistance to WLL or WLW.

Parasite line	Parental line	Selection agent	Inhibitor tested	Mean ± SEM IC_{90} (nM)[a]	N[b]	Fold change[c]	*P* value[d]
Cam3.II K13[WT]	--	--	WLL	31.7 ± 3.0	7	NA	NA
RPT4 E380*	Cam3.II K13[WT]	WLW	WLL	31.3 ± 3.4	7	1.0	0.80 (ns)
RPN6 E266K	Cam3.II K13[WT]	WLW	WLL	30.2 ± 4.5	7	1.0	>0.99 (ns)
Cam3.II K13[C580Y]	--	--	WLL	37.1 ± 5.6	6	NA	NA
β5 A20S	Cam3.II K13[C580Y]	WLL	WLL	110.5 ± 10.3	7	3.0	0.0012 (∗∗)
β2 C31Y	Cam3.II K13[C580Y]	WLW	WLL	22.3 ± 2.0	7	0.6	0.0082 (∗∗)
RPT5 G319S	Cam3.II K13[C580Y]	WLW	WLL	35.5 ± 5.6	7	1.0	0.45 (ns)
V1/S K13[WT]	--	--	WLL	32.6 ± 4.0	6	NA	NA
β6 A117V	V1/S K13[WT]	WLL	WLL	79.3 ± 9.5	7	2.4	0.0012 (∗∗)
β2 C31F	V1/S K13[WT]	WLW	WLL	18.6 ± 2.6	6	0.6	0.026 (∗)
V1/S K13[C580Y]	--	--	WLL	30.5 ± 2.8	6	NA	NA
β6 S208L	V1/S K13[C580Y]	WLL	WLL	39.3 ± 6.2	7	1.3	0.45 (ns)
β2 A49E	V1/S K13[C580Y]	WLW	WLL	33.8 ± 2.9	7	1.1	0.84 (ns)
Cam3.II K13[WT]	--	--	WLW	248.7 ± 15.3	6	NA	NA
RPT4 E380*	Cam3.II K13[WT]	WLW	WLW	424.3 ± 37.2	6	1.7	0.0043 (∗∗)
RPN6 E266K	Cam3.II K13[WT]	WLW	WLW	459.4 ± 23.9	6	1.8	0.0022 (∗∗)
Cam3.II K13[C580Y]	--	--	WLW	284.9 ± 16.8	6	NA	NA
β5 A20S	Cam3.II K13[C580Y]	WLL	WLW	86.9 ± 7.9	7	0.3	0.0012 (∗∗)
β2 C31Y	Cam3.II K13[C580Y]	WLW	WLW	615.5 ± 30.6	7	2.5	0.0012 (∗∗)
RPT5 G319S	Cam3.II K13[C580Y]	WLW	WLW	464.6 ± 43.4	5	1.9	0.017 (∗)
V1/S K13[WT]	--	--	WLW	309.0 ± 54.7	6	NA	NA
β6 A117V	V1/S K13[WT]	WLL	WLW	623.9 ± 90.7	7	2.0	0.014 (∗)
β2 C31F	V1/S K13[WT]	WLW	WLW	535.7 ± 30.8	6	1.7	0.015 (∗)
V1/S K13[C580Y]	--	--	WLW	241.0 ± 18.9	6	NA	NA
β6 S208L	V1/S K13[C580Y]	WLL	WLW	235.4 ± 54.0	7	1.0	0.53 (ns)
β2 A49E	V1/S K13[C580Y]	WLW	WLW	407.2 ± 68.3	7	1.7	0.0012 (∗∗)

[a] IC_{90} values represent concentrations at which growth was inhibited by 90% in 72 hr assays with asynchronous cultures.

[b] N, number of independent experiments (each with technical duplicates).

[c] Fold change values represent the ratio of the IC_{50} of the selected line to the IC_{90} of the parental line.

[d] Statistics were performed using Mann-Whitney *U* tests, comparing K13-mutant lines to the wild-type (WT) line in the same genetic background.

NA, not applicable (reference line); ns, not significant; ∗ $P<0.05$; ∗∗ $P<0.01$.
