# Supplementary material for: Covalent Plasmodium falciparum-selective proteasome inhibitors exhibit a low propensity for generating resistance in vitro and synergize with multiple antimalarial agents
Source: PLoS Pathog. 2019 Jun 6;15(6):e1007722. doi: 10.1371/journal.ppat.1007722 (PMC6553790; doi:10.1371/journal.ppat.1007722)
Supplement: S10 Table — (PDF) [file ppat.1007722.s012.pdf]

S10 Table. IC_{50} values of activity-based probe profiling of WLL- or WLW-resistant lines.

		Mean ± SEM IC_{50} (μM)[a]		
		WLL[b]		WLW[b]
Parasite line	Parental line	β2	β5	β2
Cam3.II K13[C580Y]	--	1.5 ± 0.2	3.1 ± 0.5	0.4 ± 0.1
β5 A20S	Cam3.II K13[C580Y]	1.5 ± 0.3	8.2 ± 1.0	0.2 ± 0.1
β2 C31Y	Cam3.II K13[C580Y]	0.7 ± 0.2	3.4 ± 0.6	45.0 ± 3.1
V1/S K13[WT]	--	2.9 ± 0.8	5.2 ± 0.3	0.7 ± 0.2
β6 A117V	V1/S K13[WT]	2.5 ± 0.5	10.6 ± 2.1	0.5 ± 0.2
β2 C31F	V1/S K13[WT]	2.3 ± 0.5	9.3 ± 1.5	10.0 ± 1.9
V1/S K13[C580Y]	--	2.0 ± 0.5	4.5 ± 0.7	0.2 ± 0.02
β6 S208L	V1/S K13[C580Y]	2.6 ± 0.7	3.0 ± 0.5	0.3 ± 0.04
β2 A49E	V1/S K13[C580Y]	4.6 ± 0.8	2.3 ± 0.5	2.7 ± 0.6

[a]Values indicate concentrations required to produce 50% reduction in activity-based probe labeling of indicated proteasome β-subunit. N=2 independent experiments. Values are shown as means ± SEM.

[b]WLL or WLW were used to pretreat the parasites across a range of concentrations for 1 hr prior to activity-based probe profiling of the parasite lysates.
