# Supplementary material for: Covalent Plasmodium falciparum-selective proteasome inhibitors exhibit a low propensity for generating resistance in vitro and synergize with multiple antimalarial agents
Source: PLoS Pathog. 2019 Jun 6;15(6):e1007722. doi: 10.1371/journal.ppat.1007722 (PMC6553790; doi:10.1371/journal.ppat.1007722)
Supplement: S11 Table — (PDF) [file ppat.1007722.s013.pdf]

S11 Table. Compounds used for isobologram analyses.

Compound	Abbreviation	Target/mode of action	Status	Source
Mu-WLL-vs	WLL	*Plasmodium*-specific proteasome inhibitor; inhibits β2 and β5 active sites	Experimental compound	Bogyo Laboratory, Stanford[a]
Mu-WLW-vs	WLW	*Plasmodium*-specific proteasome inhibitor; inhibits β2 active site	Experimental compound	Bogyo Laboratory, Stanford[a]
Dihydroartemisin	DHA	Carbon-centered radicals cause alkyation of proteins, lipids and heme	ACT core component	Avachem Scientific
OZ439	--	Suspected to be similar to DHA	In human clinical trials	Medicines for Malaria Venture
Methylene Blue	MB	Disrupts redox equilibrium	Licensed for other indications	American Regent
b-AP15	--	Inhibitor of proteasome-associated deubiquitinases	Experimental compound	Cayman Chemical
Eeyarestatin I	ES_I	ERAD inhibitor; blocks release of misfolded proteins from ER	Experimental compound	Cayman Chemical
Atovaquone	ATQ	Inhibits the cytochrome bc1 complex (mitochondrial electron transport chain)	Licensed antimalarial	Medicines for Malaria Venture
Lumefantrine	LMF	Implicated in inhibition of hemoglobin degradation and heme detoxification	ACT partner drug	Medicines for Malaria Venture
Piperaquine	PPQ	Inhibits hemoglobin degradation and heme detoxification	ACT partner drug	Avachem Scientific
Chloroquine	CQ	Inhibits hemoglobin degradation and heme detoxification	Former first-line antimalarial	Walter Reed Army Institute of Research
ACT-451840	--	Putative inhibitor of PfMDR1 that serves as resistance mediator	Experimental compound	Medicines for Malaria Venture
AN3661	--	Inhibits mRNA processing and stability factor PfCPSF3	Experimental compound	Medicines for Malaria Venture
Cyclohexamide	CHX	Inhibits elongation of nascent polypeptide chains during protein synthesis	Licensed for other indications	Cayman Chemical
DDD107498	--	Inhibits PfeEF2, important for ribosomal translocation along mRNA	Experimental compound	Medicines for Malaria Venture
DSM265	--	Inhibits PfDHODH, necessary for pyrimidine biosynthesis	Experimental compound	Medicines for Malaria Venture
Halofuginone	HFG	Inhibits cytoplasmic prolyl t-RNA synthetase	Experimental compound	Cayman Chemical
NITD609	--	Inhibits PfATP4, necessary for sodium homeostasis	Experimental compound	Medicines for Malaria Venture

[a]WLL and WLW were previously reported by Li *et al.* (2016, *Nature*).

ACT, artemisinin-based combination therapy; ERAD, ER-associated degradation; PfATP4, *P. falciparum* P-type Na+-ATPase; PfCPSF3, *P. falciparum* cleavage and polyadenylation factor-3; PfDHODH, *P. falciparum* dihydroorotate dehydrogenase; PfeEF2, *P. falciparum* translation elongation factor 2; PfMDR1, *P. falciparum* multidrug resistance gene-1.
