# Supplementary material for: Covalent Plasmodium falciparum-selective proteasome inhibitors exhibit a low propensity for generating resistance in vitro and synergize with multiple antimalarial agents
Source: PLoS Pathog. 2019 Jun 6;15(6):e1007722. doi: 10.1371/journal.ppat.1007722 (PMC6553790; doi:10.1371/journal.ppat.1007722)
Supplement: S12 Table — (PDF) [file ppat.1007722.s014.pdf]

S12 Table. Isobologram analyses of compounds tested on asynchronous parasites, synchronized rings and synchronized trophozoites, presented as the means of the fractional IC_{50} sums.

		Asynchronous				Rings				Trophozoites			
		WLL		WLW		WLL		WLW		WLL		WLW	
Drugs		Cam3.II K13^{WT}	Cam3.II K13^{C580Y}	Cam3.II K13^{WT}	Cam3.II K13^{C580Y}	Cam3.II K13^{WT}	Cam3.II K13^{C580Y}	Cam3.II K13^{WT}	Cam3.II K13^{C580Y}	Cam3.II K13^{WT}	Cam3.II K13^{C580Y}	Cam3.II K13^{WT}	Cam3.II K13^{C580Y}
DHA	Mean	0.89	1.05	0.66	0.71	0.69	0.77	0.43	0.44	0.70	0.68	0.69	0.73
	SEM	0.01	0.02	0.05	0.09	0.04	0.06	0.07	0.08	0.08	0.11	0.01	0.02
	N[a]	2	2	2	2	3	3	3	4	3	3	2	2
OZ439	Mean	1.21	1.10	0.85	0.76	0.65	0.79	0.35	0.40	0.64	0.67	0.48	0.51
	SEM	0.08	0.10	0.07	0.07	0.15	0.02	0.01	0.01	0.09	0.10	0.06	0.05
	N	3	3	3	3	2	2	2	2	5	5	5	5
MB	Mean	1.22	1.20	0.89	0.83	0.87	0.84	0.40	0.36	0.98	1.01	0.71	0.78
	SEM	0.06	0.02	0.02	0.03	0.03	0.06	0.05	0.03	0.04	0.03	0.06	0.11
	N	3	3	3	3	4	4	4	4	3	3	3	3
b-AP15	Mean	1.05	1.11	0.86	0.78	0.80	0.77	0.34	0.35	0.67	0.66	0.60	0.52
	SEM	0.02	0.07	0.04	0.06	0.08	0.05	0.11	0.06	0.02	0.06	0.01	0.03
	N	3	3	2	2	3	4	2	2	2	2	2	2
ES_I	Mean	1.28	1.29	0.98	0.97	1.05	1.09	0.73	0.80	1.19	1.05	0.97	1.08
	SEM	0.04	0.03	0.08	0.08	0.001	0.03	0.004	0.02	0.10	0.17	0.05	0.05
	N	3	3	2	2	3	3	2	2	2	2	3	3
ATQ	Mean	1.33	1.22	1.28	1.16	1.64	1.54	1.17	1.20	1.11	0.99	1.02	1.07
	SEM	0.14	0.06	0.07	0.01	0.01	0.10	0.09	0.10	0.05	0.08	0.01	0.01
	N	3	3	3	3	2	3	2	3	3	3	3	2
LMF	Mean	1.42	1.24	1.32	1.07	1.23	1.13	0.95	0.98	1.12	1.24	1.16	1.01
	SEM	0.17	0.08	0.20	0.21	0.05	0.05	0.09	0.03	0.02	0.04	0.19	0.01
	N	3	3	3	3	3	3	3	3	3	3	2	2
PPQ	Mean	1.52	1.37	1.44	1.33	1.46	1.41	1.20	1.30	1.32	1.23	1.14	1.20
	SEM	0.07	0.002	0.04	0.0004	0.03	0.06	0.07	0.12	0.01	0.04	0.09	0.09
	N	2	2	2	2	3	3	3	3	3	3	2	2
CQ	Mean	1.41	1.53	1.29	1.36	1.69	1.70	1.45	1.54	1.89	1.75	1.57	1.41
	SEM	0.10	0.17	0.07	0.04	0.31	0.02	0.20	0.02	0.10	0.12	0.12	0.13
	N	2	2	2	2	2	2	2	2	2	3	2	3

[a]N = number of independent experiments (each with technical duplicates).

ATQ, atovaquone; CQ, chloroquine; DHA, dihydroartemisinin; ES_I, eeyarestatin I; LMF, lumefantrine; MB, methylene blue; PPQ, piperaquine.
