# Supplementary material for: Covalent Plasmodium falciparum-selective proteasome inhibitors exhibit a low propensity for generating resistance in vitro and synergize with multiple antimalarial agents
Source: PLoS Pathog. 2019 Jun 6;15(6):e1007722. doi: 10.1371/journal.ppat.1007722 (PMC6553790; doi:10.1371/journal.ppat.1007722)
Supplement: S13 Table — (PDF) [file ppat.1007722.s015.pdf]

S13 Table. Fractional IC$_{50}$ values from isobologram analyses on asynchronous parasites only, presented as the means of the FIC$_{50}$ sums.

Drugs		Asynchronous			
		WLL		WLW	
		Cam3.II K13^{WT}	Cam3.II K13^{C580Y}	Cam3.II K13^{WT}	Cam3.II K13^{C580Y}
ACT-451840	Mean	1.34	1.27	1.16	1.14
	SEM	0.003	0.02	0.02	0.02
	N	2	2	2	2
AN3661	Mean	1.69	1.58	1.64	1.63
	SEM	0.04	0.0005	0.01	0.08
	N	2	2	2	2
CHX	Mean	1.44	1.40	1.29	1.37
	SEM	0.05	0.11	0.07	0.08
	N	3	3	3	3
DDD 107498	Mean	1.65	1.60	1.28	1.44
	SEM	0.11	0.11	0.13	0.16
	N	2	2	2	2
DSM265	Mean	1.34	1.46	1.24	1.30
	SEM	0.08	0.05	0.02	0.03
	N	2	2	2	2
HFG	Mean	1.47	1.46	1.42	1.53
	SEM	0.001	0.01	0.05	0.04
	N	2	2	2	2
NITD609	Mean	1.58	1.67	1.39	1.44
	SEM	0.07	0.10	0.03	0.05
	N	2	2	2	2

[a]N = number of independent experiments (each with technical duplicates).

CHX, Cyclohexamide; HFG, halofuginone.
